# Dual-Scale Hybrid Concept Bottleneck Network for Explainable 3D Lung Nodule Malignancy Classification in CT Imaging

**DOI:** 10.3390/jimaging12090455

**Published:** 2026-09-20

**Authors:** Ahmad Ali, Muhammad Aksam Iftikhar, Ghulam Farooque, Allah Bux Sargano

**Affiliations:** 1 Department of Computer Science, COMSATS University Islamabad, Lahore 54000, Pakistan; ahmedalisargana@gmail.com; 2 Department of Artificial Intelligence, The University of Lahore, Lahore 54000, Pakistan; 3 Department of Computer Science and Information Technology, The University of Lahore, Lahore 54000, Pakistan; ghulam.farooque@cs.uol.edu.pk; 4 Department of Linguistics, Literary and Aesthetic Studies, University of Bergen, 5007 Bergen, Norway

**Keywords:** lung nodule malignancy classification, computed tomography, explainable artificial intelligence, 3D deep learning, hybrid concept bottleneck, cross-attention fusion, Transformer, external validation

## Abstract

Accurate differentiation of benign and malignant lung nodules in computed tomography (CT) is important for early lung cancer diagnosis and reliable clinical decision-making. Many existing deep learning methods emphasize either nodule-centred morphology or broader anatomical context and provide limited insight into the radiological information represented by the model. This study proposes a Dual-Scale Hybrid Concept Bottleneck Network (DS-HCBN) for explainable 3D lung nodule malignancy classification. The framework processes a local nodule-centred patch and a larger contextual patch using a shared residual 3D convolutional encoder and a lightweight contextual Transformer. The resulting representations are integrated through gated cross-attention, while eight radiological attributes are learned as supervised intermediate representations within the hybrid classifier. The model was developed on LIDC-IDRI using a leakage-controlled patient-wise split and evaluated on a held-out internal test set. External evaluation was performed on the publicly annotated LNDb cohort using the same frozen model without retraining or fine-tuning. On the LIDC-IDRI internal test set, DS-HCBN achieved 87.34% accuracy, an F1-score of 80.39%, and a ROC-AUC of 94.35%. On LNDb, the model achieved 80.41% accuracy and a ROC-AUC of 78.65%. These results show strong internal discrimination but a clear reduction in cross-dataset performance. The findings support the use of local morphology, anatomical context, and radiological concept supervision for concept-guided lung nodule classification, while highlighting the need for improved domain generalization before broader clinical application.

## 1. Introduction

Lung nodule malignancy classification is an important clinical task for early lung cancer diagnosis and patient management. Lung cancer remains one of the leading causes of cancer-related mortality worldwide, and early detection is essential for improving survival outcomes and reducing disease burden [1,2]. Pulmonary nodules are small focal lesions within the lung parenchyma, typically ranging from 3 mm to 30 mm in diameter, and may represent either benign abnormalities or malignant tumours. Accurate malignancy assessment is clinically important because suspicious nodules require timely follow-up, diagnostic work-up, and treatment planning, whereas benign nodules may avoid unnecessary invasive procedures, healthcare costs, and patient anxiety [3,4].

Computed tomography (CT) is the primary imaging modality used for lung nodule assessment because it provides detailed cross-sectional and volumetric information about pulmonary structures. Each CT voxel represents tissue density in Hounsfield units, allowing radiologists to evaluate nodule characteristics such as size, shape, margin definition, density, calcification, texture, spiculation, and lobulation. Unlike two-dimensional imaging modalities, CT enables the assessment of nodules across the full three-dimensional volume, allowing morphological characteristics to be examined throughout the lesion. Furthermore, the growing adoption of low-dose CT screening programmes has increased the need for reliable computer-assisted tools that can support large-scale lung cancer screening while maintaining diagnostic accuracy and efficiency [5].

Despite its diagnostic value, manual CT interpretation remains time-consuming, subject to inter-observer variability, and dependent on radiological expertise. Consequently, deep learning methods have become increasingly important for computer-assisted lung nodule analysis. Three-dimensional convolutional neural networks (3D CNNs) have demonstrated strong capability for learning volumetric representations of nodule morphology, while Transformer-based architectures have shown promise in modelling long-range spatial dependencies and contextual information. However, many existing models operate as black boxes whose predictions are difficult to interpret, which may limit their suitability for transparent clinical decision-support workflows [6,7].

To address concerns regarding model transparency, explainable artificial intelligence (XAI) techniques have been increasingly adopted in medical imaging. Popular post-hoc approaches such as Grad-CAM and SHAP can highlight image regions or features associated with model predictions [8,9]. While these methods can provide useful complementary explanations, they do not explicitly incorporate clinically meaningful radiological knowledge into the prediction pathway. Consequently, they may indicate where a model responds without directly describing which radiological characteristics are represented within the learned decision process.

An alternative approach is concept bottleneck learning, which introduces human-interpretable intermediate concepts between image features and diagnostic predictions. In lung nodule assessment, radiological characteristics such as subtlety, sphericity, margin definition, lobulation, spiculation, texture, internal structure, and calcification provide a natural set of clinically meaningful attributes that are routinely assessed by radiologists. Because the LIDC-IDRI dataset contains reader-provided assessments of these characteristics, it offers a valuable opportunity to investigate concept-guided learning in three-dimensional CT analysis.

A strict concept bottleneck model derives the final prediction exclusively from predicted concepts. Such a formulation provides a transparent decision pathway, but its performance may be constrained when the predefined concepts are noisy, incompletely annotated, or insufficient to represent all relevant imaging evidence. The present study therefore adopts a hybrid concept bottleneck formulation, in which predicted radiological concepts provide an explicit, interpretable pathway while latent imaging features remain available to the malignancy classifier. This design aims to balance predictive representation capacity and semantic interpretability. Because latent features remain available, however, the predicted concepts should not be interpreted as a complete causal explanation of every model decision. Their predictive accuracy and contribution to malignancy classification must therefore be evaluated quantitatively.

Although previous studies have explored 3D CNNs, Transformer-based architectures, attention mechanisms, and post-hoc explanation methods for lung nodule malignancy classification, several limitations remain. Many methods rely primarily on either small lesion-centred regions or larger contextual regions and therefore may not fully exploit complementary information available at different spatial scales. Furthermore, most studies focus predominantly on predictive performance and provide limited insight into the radiological attributes represented by the model. Independent external validation is also relatively uncommon despite its importance for assessing cross-dataset generalization under differences in acquisition protocols, patient populations, annotation practices, and class distributions. Consequently, the combined use of dual-scale volumetric representation, radiological concept supervision, and cross-dataset evaluation remains insufficiently explored in lung nodule classification.

To address these limitations, this study proposes a Dual-Scale Hybrid Concept Bottleneck Network (DS-HCBN) for nodule-level malignancy classification from CT images. The framework jointly analyses a local nodule-centred patch and a larger contextual patch to capture complementary information from lesion morphology and surrounding anatomy. A shared residual 3D convolutional encoder provides the primary morphology representation, while a lightweight Transformer models the larger contextual volume. Contextual information is incorporated through learned residual gating and local-anchored cross-attention. In parallel, the model predicts eight radiological attributes using their native annotation structures: six ordinal concepts and two categorical concepts. The resulting concept representation contributes to malignancy classification together with the latent imaging representation through a hybrid concept-guided pathway.

The proposed system specifically addresses malignancy classification of pre-identified and localized pulmonary nodules. It assumes that a nodule centroid is available for extraction of the local and contextual patches and does not perform nodule detection or localization from complete clinical CT volumes. Consequently, the reported classification performance should not be interpreted as end-to-end lung cancer screening or diagnostic performance. In addition, the primary binary task uses benign and malignant nodules defined from radiologist malignancy assessments, while nodules with intermediate reader-averaged scores are excluded from the binary endpoint. This produces a clearly defined binary classification task but does not address the full spectrum of clinically ambiguous nodules, which remains an important limitation and direction for future work.

The main contributions of this study are summarised as follows:

- A dual-scale 3D framework is proposed for pulmonary nodule malignancy classification that jointly analyses a local $64^{3}$ nodule-centred volume and a larger $96^{3}$ contextual volume to represent complementary lesion morphology and surrounding anatomical information.
- A hybrid CNN–Transformer architecture is developed in which a shared residual 3D convolutional encoder provides the primary morphology representation, a lightweight contextual Transformer captures longer-range information, and local-anchored gated cross-attention integrates the two representations while controlling the contribution of contextual information.
- A hybrid concept bottleneck formulation incorporates eight radiologist-defined nodule characteristics while preserving their native annotation structures: six attributes are modelled as ordinal variables and internal structure and calcification are modelled as categorical variables. Multi-reader disagreement is retained through reader-level soft supervision rather than reducing all concept annotations to averaged hard labels.
- A leakage-controlled patient-wise evaluation protocol is employed in which data partitioning precedes augmentation, model and operating-threshold selection are performed using validation data only, and the held-out internal test set is used only after model freezing. Statistical uncertainty, calibration, concept-level behaviour, and cross-dataset generalization are evaluated to provide a broader assessment than classification accuracy alone.

Rather than interpreting strong internal classification performance as evidence of clinical readiness, this study evaluates the proposed model in terms of discrimination, uncertainty, interpretability, and cross-dataset generalization. External evaluation on LNDb is used to investigate the extent to which performance transfers to data acquired and annotated independently from LIDC-IDRI; observed cross-dataset differences are analysed rather than being interpreted as evidence of universal robustness.

The rest of the paper is organised as follows. Section 2 reviews related work on lung nodule classification and explainable medical AI. Section 3 describes the proposed methodology, including preprocessing, dual-scale representation learning, the hybrid concept bottleneck formulation, and the training objective. Section 4 presents the experimental evaluation, including internal testing, ablation analysis, explainability assessment, and external validation. Section 5 discusses the findings, limitations, and implications, and Section 6 concludes the paper with future research directions.

## 2. Literature Review

Deep learning has substantially advanced automated analysis of thoracic computed tomography (CT), including pulmonary nodule detection, segmentation, and malignancy classification. Although these tasks are related, they address different stages of the diagnostic pipeline and should not be treated as interchangeable. Detection identifies candidate nodules, segmentation delineates their boundaries, whereas malignancy classification estimates the likelihood of benign or malignant pathology for an already identified lesion. The present work focuses specifically on nodule-level malignancy classification using pre-localized 3D CT volumes.

Existing studies differ considerably in input representation, dataset construction, label definition, splitting strategy, and evaluation protocol. Consequently, reported performance values should be interpreted in the context of the corresponding experimental setting. Results obtained from individual 2D slices, complete CT scans, chest radiographs, or multimodal disease datasets are not directly comparable with those obtained from nodule-centred 3D volumes. Likewise, patient-wise evaluation provides a more stringent setting than image-level random splitting when multiple slices or nodules can originate from the same patient. The following review therefore considers not only predictive performance but also how successive methodological developments address volumetric representation, contextual modelling, interpretability, and generalization.

### 2.1. 2D Lung Nodule Classification Methods

Early deep-learning approaches to pulmonary nodule classification were dominated by two-dimensional convolutional models, largely because mature CNN architectures and large-scale pretrained weights were readily available. Transfer-learning pipelines based on VGGNet [10], AlexNet [11], GoogLeNet [12], DenseNet [13], ResNet [14], and related architectures demonstrated that discriminative malignancy features could be extracted efficiently from individual CT slices. For example, combinations of convolutional and capsule representations achieved high reported performance on LIDC-IDRI, including an AUC of 98.0% and an accuracy of 98.61% [15], while ensemble CNN approaches reported approximately 95% accuracy on LUNA16 [16]. Hybrid schemes in which pretrained CNNs served as feature extractors for conventional classifiers also produced strong results; AlexNet/GoogLeNet features combined with an SVM, for instance, yielded very high precision and specificity in the evaluated LIDC-IDRI setting [17].

These results established the usefulness of learned image representations, but they also revealed an important representational limitation. A pulmonary nodule is inherently a three-dimensional structure, and features associated with malignancy—including spiculation, lobulation, margin irregularity, calcification pattern, and heterogeneous texture—may extend across several neighbouring slices. Processing an isolated slice therefore sacrifices inter-slice continuity and can make the learned representation dependent on slice selection. Evaluations across more than one dataset, such as the modified VGG approach reporting 92.6% accuracy on LIDC and 97.0% on NLST [18], provide useful evidence of transferability, but the underlying 2D representation still cannot explicitly encode the complete spatial morphology of the lesion. This limitation motivated a transition from slice-based classification toward volumetric learning, where spatial relationships across all three axes can be represented directly.

### 2.2. 3D CT-Based Nodule Classification Methods

Three-dimensional CNNs extend convolution to volumetric neighbourhoods and therefore provide a more natural representation for pulmonary nodules. Initial 3D frameworks often combined segmentation and classification so that lesion boundaries and malignancy-related features could be learned within the same pipeline. Architectures integrating 3D-VNet and 3D-ResNet, for example, reported 99.2% accuracy, 98.8% sensitivity, and 99.6% specificity on LUNA16 [19]. Although such results demonstrate the capacity of volumetric learning, detection- or segmentation-oriented pipelines solve a broader task than the pre-localized nodule classification setting considered here.

As volumetric CNNs matured, attention and multi-scale receptive fields were introduced to improve sensitivity to heterogeneous nodule morphology. A multi-scale 3D CNN with attention achieved 98.7% accuracy and a 96.8% F1-score on LUNA16 [20], illustrating the value of emphasizing informative spatial features at different receptive-field sizes. However, attention weights remain latent importance measures rather than explicit radiological explanations. Other work has instead focused on representation learning and transfer. Self-supervised 3D learning combined with domain adaptation achieved 91.07% accuracy and 95.84% AUC on LIDC-IDRI [21], highlighting the potential of unlabeled CT data for improving feature generalization, while leaving semantic interpretability largely unaddressed.

A further development has been to represent the nodule through complementary spatial views. Multi-view 3D convolution with squeeze-and-excitation mechanisms produced 96.04% accuracy and 98.59% sensitivity on LIDC-IDRI [22]. This approach is relevant because it demonstrates that information from multiple spatial representations can improve classification. Nevertheless, multi-view feature aggregation differs conceptually from explicitly separating a high-resolution nodule-centred volume from a larger anatomical context and allowing the interaction between those representations to be learned.

More integrated volumetric pipelines have also appeared. 3D-NoduleNet [23], which incorporates 3D Faster R-CNN and 3D BCDU-Net components, reported 91.50% accuracy and a 90.63% F1-score on LIDC-IDRI/LUNA16. Its broader design addresses spatial localization and segmentation-related processing in addition to classification. In contrast, the present study deliberately assumes that the nodule has already been localized and investigates how its fine morphology and surrounding volumetric context can be jointly represented for malignancy prediction. The distinction is therefore one of task formulation and representation rather than an assertion that one architecture is universally superior.

Taken together, 3D methods address the principal spatial limitation of 2D approaches, but high-quality volumetric representation alone does not resolve how local lesion detail should be combined with broader context, nor does it provide an intrinsically semantic explanation of the resulting decision. These issues motivated the subsequent use of Transformer-based contextual modelling and hybrid feature-learning architectures.

### 2.3. Hybrid CNN-Transformer and Attention-Based Methods

CNNs and Transformers provide complementary inductive biases. Convolutional networks are particularly effective at capturing local spatial patterns, whereas self-attention can model relationships over larger spatial extents. In pulmonary nodule analysis, this complementarity has encouraged hybrid architectures in which convolutional morphology features are augmented with contextual, attribute-based, or globally aggregated representations.

Before the widespread adoption of Transformers, similar objectives were pursued by combining handcrafted morphological or texture descriptors with deep convolutional features. Such mixed-feature models achieved competitive performance on LIDC-IDRI; for example, a combination of engineered descriptors and CNN representations obtained 91.21% accuracy and a 91.04% F1-score [24]. These methods demonstrated that complementary feature spaces can improve malignancy classification, but handcrafted descriptors require explicit feature design and do not provide the same end-to-end interaction between local and contextual volumetric features.

Transformer-based approaches subsequently provided a more flexible mechanism for modelling long-range dependencies. Nodule-CLIP [25], for example, combines CT representations with radiologist-annotated attributes in a multimodal contrastive framework using a ResNet-34 image representation and a Transformer-based attribute encoder. Its reported accuracy of 90.6% and recall of 92.81% on LIDC-IDRI show that semantic attributes can complement image features. Importantly, however, the role of attributes in Nodule-CLIP differs from their role in a concept-bottleneck model: they participate in multimodal representation learning, whereas in the present framework radiological concepts are predicted as explicit intermediate outputs from CT and are allowed to contribute directly to the malignancy decision.

Other CNN–attention, recurrent, and Transformer hybrids have similarly demonstrated the benefit of combining heterogeneous representations for lung-related image classification [26,27,28]. However, many of these methods operate on disease-level, multimodal, or image-level settings rather than localized 3D pulmonary nodules. Their results therefore support the general architectural motivation for hybrid learning but cannot serve as direct numerical benchmarks for the present task.

The relevant methodological progression is therefore not simply from CNNs to Transformers, but from isolated feature extractors toward architectures in which local and contextual representations interact explicitly. The proposed DS-HCBN follows this direction by retaining convolutional morphology as the primary representation, modelling the larger contextual volume with a lightweight Transformer, and allowing local features to attend selectively to contextual tokens through gated cross-attention. This differs from simple concatenation because the interaction between feature spaces is content-dependent rather than fixed. It also differs from attribute-aware multimodal models such as Nodule-CLIP because the semantic concepts are predicted within the same CT-based classification pathway.

### 2.4. Explainable AI and Concept Bottleneck Methods

As predictive architectures have become more complex, interpretability has emerged as a parallel concern in medical imaging. The most common strategy has been post-hoc explanation, where an already trained classifier is analysed after prediction. Spatial methods such as Grad-CAM can identify image regions associated with a decision, while LIME and SHAP estimate local or feature-level contributions. In lung-related imaging, these approaches have been used with convolutional, transfer-learning, and hybrid classifiers [5,29,30,31,32].

Post-hoc visualization improves transparency, but it does not necessarily reveal which radiological property drives a prediction. A heatmap concentrated around a nodule boundary, for instance, does not distinguish whether the model responded to spiculation, lobulation, margin irregularity, or texture. Furthermore, visual plausibility should not be equated with faithfulness: an explanation may appear clinically sensible without accurately reflecting the internal decision mechanism.

This limitation has encouraged models that integrate semantic supervision directly into learning. Multi-task pulmonary nodule frameworks have combined malignancy prediction with manifestation identification and anatomical attention, achieving AUC values close to 0.992 on LIDC-IDRI and 0.923 on an independent institutional dataset [33]. Such models move beyond pure saliency by predicting radiologically meaningful intermediate information, although auxiliary semantic tasks do not necessarily constitute a concept bottleneck unless those intermediate variables form an explicit representation used by the final prediction.

Concept bottleneck models make this relationship more explicit by introducing human-understandable variables between the input and task output. For pulmonary nodules, the LIDC-IDRI semantic ratings: subtlety, internal structure, calcification, sphericity, margin, lobulation, spiculation, and texture provide a natural concept space. Expert-driven concept frameworks such as ClinicXAI [34] demonstrate the broader value of semantic intermediate representations for explainable medical AI, although its chest-radiograph setting differs from volumetric CT nodule classification.

Concept-based learning also introduces an important methodological requirement: interpretability cannot be inferred merely from the existence of concept outputs. The concepts must first be predicted with sufficient fidelity, and the downstream classifier must demonstrably depend on them. This issue is particularly important for a hybrid concept bottleneck, where latent imaging features remain available to the malignancy classifier. A hybrid formulation may improve predictive flexibility, but the latent pathway can potentially bypass the semantic representation. Quantitative concept evaluation and intervention analysis are therefore necessary to determine whether the concept pathway contributes meaningful information rather than serving only as an auxiliary output.

Viewed together, the literature shows a clear methodological progression: 2D CNNs established the feasibility of learned CT representations; 3D architectures preserved volumetric morphology; attention and Transformer-based models extended the effective spatial context; and XAI and concept-based learning introduced increasingly explicit forms of interpretability. However, these advances have generally been investigated in separate formulations. Volumetric models often emphasize discrimination without semantic reasoning, attribute-aware approaches do not necessarily use predicted concepts as intermediate decision variables, and post-hoc explanations may remain visually plausible without being faithful.

The proposed DS-HCBN is positioned at the intersection of these directions. It combines a nodule-centred local volume with a larger contextual volume, extracts morphology using a shared residual 3D CNN, models contextual tokens using a lightweight Transformer, and performs gated local-to-context cross-attention. The resulting latent representation is paired with six ordinal and two categorical radiological concepts within a hybrid concept-guided malignancy head. The contribution therefore lies in the integration of dual-scale volumetric representation, selective contextual fusion, and radiological concept supervision rather than in claiming novelty for CNNs, Transformers, attention, or concept learning individually.

Because published studies differ considerably in task definition, dataset composition, and validation design, their reported metrics should be regarded as contextual rather than as a controlled ranking. Table 1 therefore summarizes the closest methods in terms of representation, task scope, interpretability mechanism, and principal methodological distinction from DS-HCBN.

## 3. Proposed Methodology

**Figure 1:**
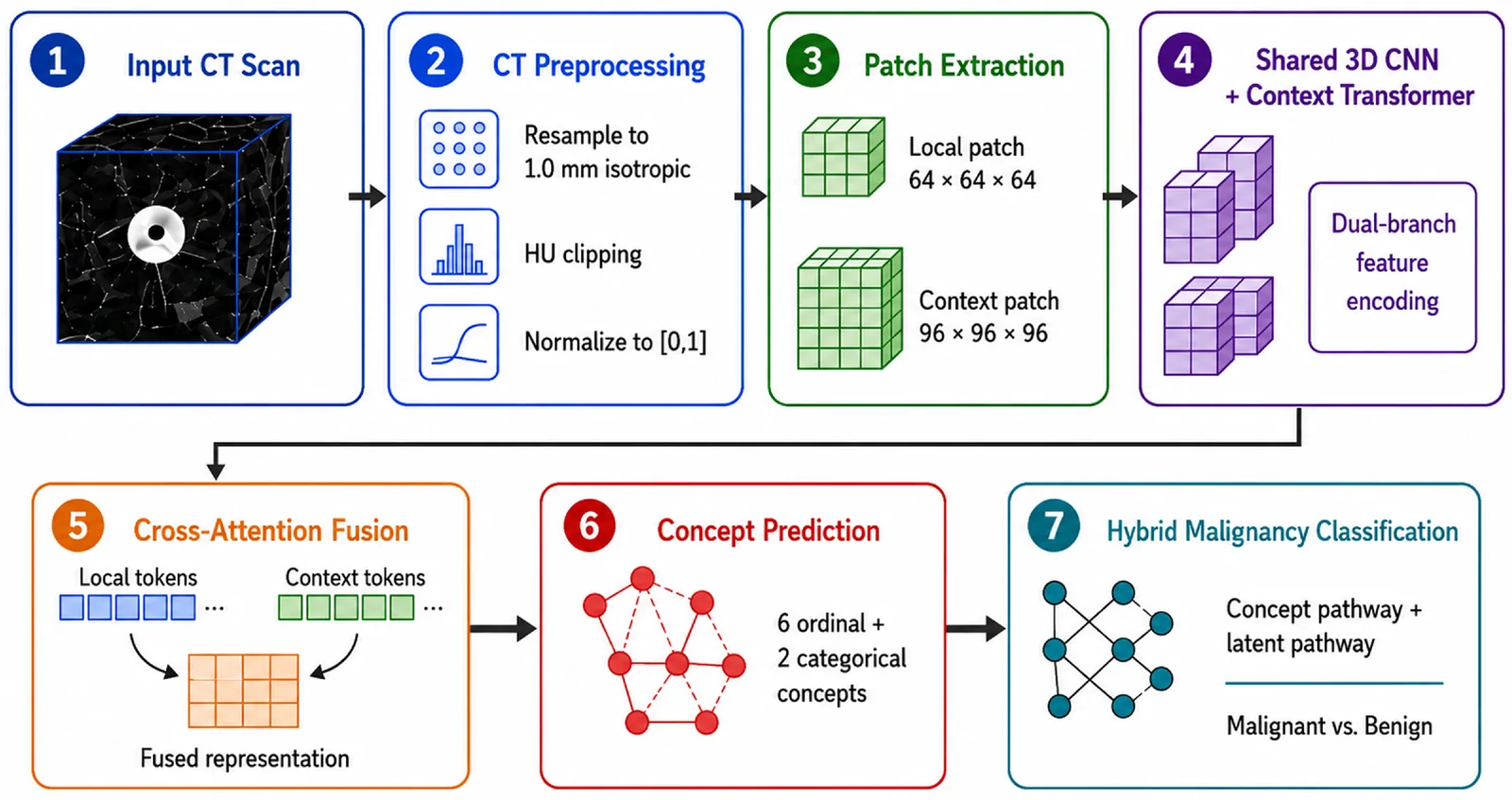
Workflow of the proposed dual-scale 3D lung nodule classification framework.

**Figure 2:**
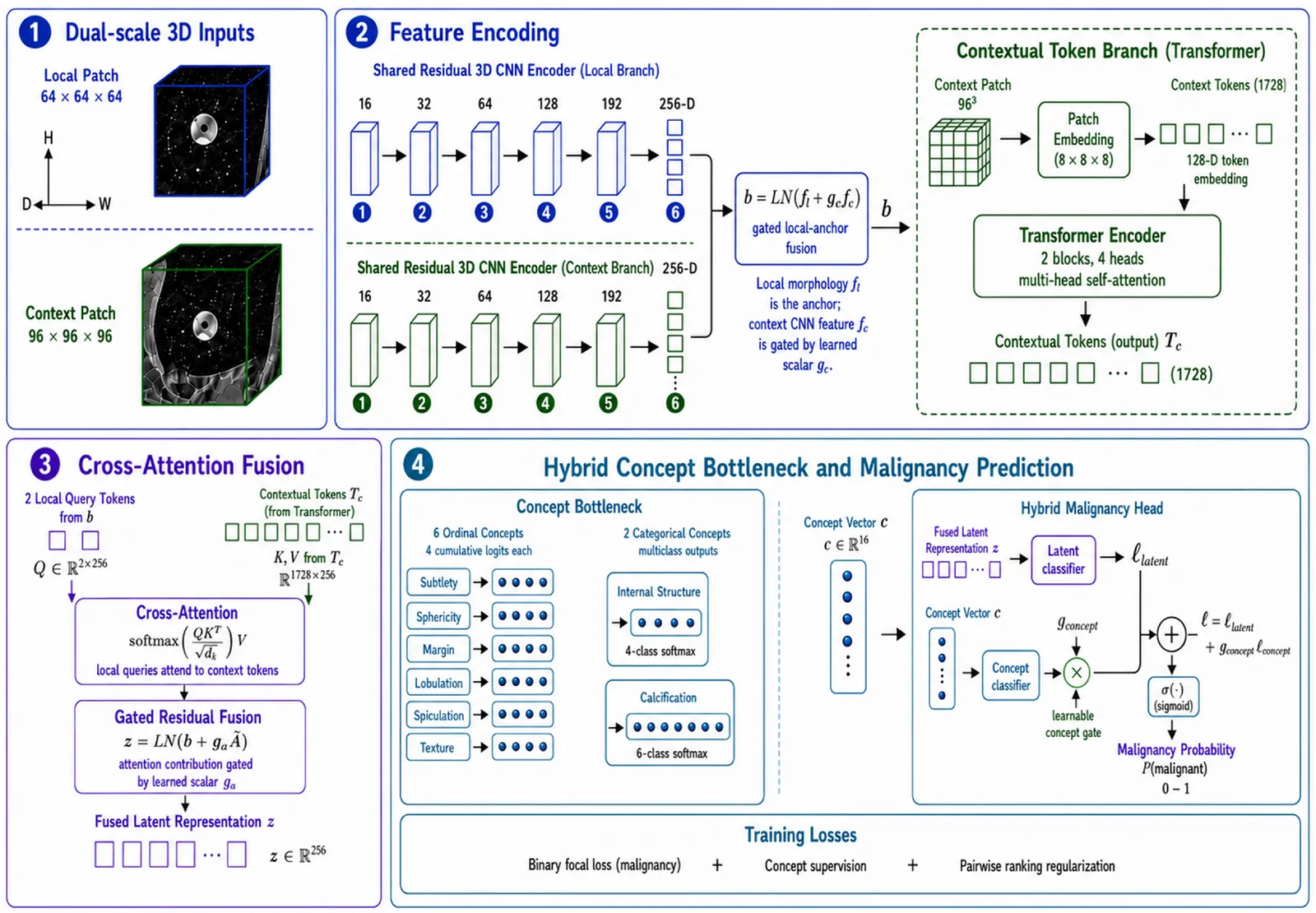
Architecture of the proposed Dual-Scale Hybrid Concept Bottleneck Network (DS-HCBN). Local and contextual CT patches are processed by a shared residual 3D convolutional morphology encoder. The contextual patch is additionally represented by a lightweight Transformer. Contextual information is introduced into the local morphology representation through two learned residual gates and cross-attention. The resulting fused representation is used by the radiological concept heads and the malignancy classifier.

The proposed methodology combines dual-scale volumetric analysis, residual 3D convolutional feature learning, contextual Transformer modelling, gated cross-attention, and radiological concept supervision for malignancy classification of pre-localized pulmonary nodules. Figure 1 presents the overall workflow, and Figure 2 shows the detailed network architecture.

### 3.1. Dataset Preparation and Preprocessing

The LIDC-IDRI dataset [35] was used for model development and internal evaluation. It provides thoracic CT scans with independent annotations from multiple radiologists, including malignancy ratings and the radiological attributes used for concept supervision. The task is nodule-level benign/malignant classification of pre-identified pulmonary nodules; a nodule centroid is therefore assumed to be available.

A consistent preprocessing pipeline was applied to all CT scans. Non-diagnostic localizer series and scans with fewer than 50 slices were excluded. When multiple CT series were available for a patient, the series referenced by the corresponding LIDC XML annotation was identified using the SeriesInstanceUID, ensuring that the processed volume matched the annotated series. DICOM images were converted to volumetric form using SimpleITK while preserving voxel spacing, origin, orientation, and slice geometry.

CT intensities were clipped to the Hounsfield unit range [−1000,400] and linearly normalized to [0,1]

(1) $$I_{\mathrm{clip}}=\min\left(\max(I,-1000),400\right),$$

(2) $$I_{\mathrm{norm}}=\frac{I_{\mathrm{clip}}+1000}{1400}.$$

All volumes were resampled with linear interpolation to $1.0\times 1.0\times 1.0\;{\mathrm{mm}}^{3}$ isotropic spacing so that a fixed voxel crop represented a comparable physical field of view across scans. No explicit denoising filter was applied because smoothing can suppress fine nodule-boundary and texture information. Robustness to modest intensity variation and noise was instead encouraged through training-only augmentation. Annotations from different radiologists were associated in 3D physical space using a constrained complete-linkage rule with a maximum centroid separation of 10mm.

A group was accepted only if every pair of annotation centroids met this distance criterion, and no group could contain more than one annotation from the same radiologist. These constraints reduce the risk of merging two nearby nodules through a single-linkage chain. The 10mm value was used as an annotation-association threshold rather than a definition of nodule extent. Its robustness was subsequently examined by reconstructing the annotation groups at 5, 7.5, 10, 12.5 and 15 mm, as described below. The group centroid was computed from the contributing reader annotations and used for patch extraction.

The reader-averaged malignancy score was used only to construct the binary endpoint; radiological concept annotations were retained at the individual-reader level. Subtlety, sphericity, margin, lobulation, spiculation, and texture were treated as ordinal attributes with ratings from 1 to 5, whereas internal structure and calcification were treated as categorical attributes with four and six valid classes, respectively. Out-of-range concept ratings were excluded during preprocessing. Reader-level concept targets are described in Section 3.3. For the binary task, nodules with mean malignancy ≤2 were labelled benign, nodules with mean malignancy ≥4 were labelled malignant, and scores strictly between 2 and 4 were treated as uncertain and excluded. Table 2 summarizes the rule.

Preprocessing produced 2664 annotated nodules from 875 patients: 793 met the benign criterion, 313 met the malignant criterion, and 1558 were uncertain. The final binary cohort therefore contained 1106 nodules from 589 patients. Thus, 1558 of 2664 nodules (58.5%) were excluded from the primary binary endpoint, which produces a clearly defined binary task but limits conclusions for clinically ambiguous nodules. Nodules with a single valid reader annotation were retained when they met the endpoint criteria and were examined later in the error analysis.

To prevent patient-level leakage, all nodules from a patient were assigned to one subset. A fixed patient-level stratified-random split was generated with seed 42 using only pre-model characteristics: the presence of malignant nodules, malignant-nodule burden, and the presence of single-reader malignant annotations. No model prediction, confidence, or classification error was used during split construction. The resulting distribution is shown in Table 3.

The split was frozen before augmentation. Augmentation was applied on-the-fly only to training samples, with the same spatial transform applied to paired local and contextual patches. It comprised random axis flips, $90^{\circ }$ rotations, intensity shifts up to ±0.015, and Gaussian noise with σ≤0.010. Class imbalance was handled with weighted sampling in the training subset rather than a fixed synthetic expansion.

For each nodule, local $64^{3}$ and contextual $96^{3}$ voxel patches were extracted around the physical centroid obtained from the corresponding multi-reader annotation group. At 1-mm isotropic spacing, these patches represent approximately $64\times 64\times 64\;{\mathrm{mm}}^{3}$ and $96\times 96\times 96\;{\mathrm{mm}}^{3}$ fields of view, respectively. The local patch emphasizes lesion morphology and boundary characteristics, whereas the contextual patch includes a larger region of surrounding anatomy.

The 10mm criterion described above was used only to associate spatially close annotations from different radiologists that were likely to refer to the same underlying lesion; it was not used to define nodule size, segmentation extent, malignancy status, or the dimensions of the extracted patches. The constrained complete-linkage requirement, together with the restriction that a radiologist could contribute at most one annotation to a nodule group, reduces the risk of merging nearby but distinct lesions. A data-level sensitivity analysis varied this threshold without changing the image-processing, label-aggregation, or modelling procedures.

### 3.2. Dual-Scale Hybrid Concept Bottleneck Network (DS-HCBN)

DS-HCBN combines a shared residual 3D CNN, a context-only Transformer, local-anchored gated cross-attention, and a hybrid concept-guided malignancy head. For each nodule, the local input is $x_{l}\in R^{64\times 64\times 64}$ and the contextual input is $x_{c}\in R^{96\times 96\times 96}$. Figure 2 summarizes the four main components.

#### 3.2.1. Dual-Scale Residual 3D Morphology Encoder

Both inputs are processed by the same residual 3D CNN, $E_{\mathrm{CNN}}\left(\cdot \right)$, with shared weights

(3) $$f_{l}=E_{\mathrm{CNN}}\left(x_{l}\right),\;f_{c}=E_{\mathrm{CNN}}\left(x_{c}\right),$$

where $f_{l},f_{c}\in R^{256}$. Weight sharing reduces parameters and encourages scale-consistent morphology features while the two crops retain different physical fields of view.

The encoder begins with a 3×3×3 convolution producing 16 channels, followed by four residual downsampling blocks with 32, 64, 128, and 192 channels. Each block contains two 3×3×3 convolutions, Group Normalization, and SiLU activation. The first convolution uses stride 2 for downsampling, and a 1×1×1 residual projection is used when the channel dimension changes. Dropout3D with rate 0.05 is applied within the residual blocks. Group Normalization is used because the 3D inputs restrict training to small mini-batches and therefore make batch-dependent statistics less stable.

Adaptive global average pooling followed by a linear projection, Layer Normalization, and SiLU activation produces the 256-D features in Equation (3). The local morphology feature remains the primary anchor, while the contextual CNN feature is introduced through a learned residual gate

(4) $$b=\mathrm{LN}\left(f_{l}+g_{c}f_{c}\right),\;g_{c}=\sigma \left(a_{c}\right).$$

The scalar gate is initialized so that $g_{c}\approx 0.10$, allowing optimization to begin close to a local morphology representation while learning how much contextual CNN information to retain.

#### 3.2.2. Contextual Transformer Representation

In parallel, the $96^{3}$ contextual patch is processed by a lightweight Transformer. It is partitioned into non-overlapping $8^{3}$ volumetric patches using a strided 3D convolution, producing

(5) $$N_{c}={\left(\frac{96}{8}\right)}^{3}=12^{3}=1728$$

tokens. Each token is projected to 128 dimensions, and fixed 3D sinusoidal positional encodings are added

(6) $$T_{0}=P\left(x_{c}\right)+P_{3D}.$$

Two Transformer encoder blocks, each with four-head self-attention, feed-forward ratio 2, Layer Normalization, residual connections, GELU, and dropout 0.15, produce

(7) $$T_{c}=E_{T}\left(T_{0}\right),\;T_{c}\in R^{1728\times 128}.$$

#### 3.2.3. Local-Anchored Cross-Attention Fusion

The Transformer representation is integrated with the gated morphology feature b. A small query-projection network maps b to two 256-D local query tokens

(8) $$Q_{0}=\mathrm{reshape}\left(\phi_{Q}\left(b\right)\right)\in R^{2\times 256},$$

where $\phi_{Q}\left(\cdot \right)$ comprises a linear projection followed by SiLU and dropout. The local queries and 128-D contextual tokens are projected into the same 256-D attention space

(9) $$Q=Q_{0}W_{Q},\;K=T_{c}W_{K},\;V=T_{c}W_{V}.$$

Thus, $Q\in R^{2\times 256}$ and $K,V\in R^{1728\times 256}$. Four-head cross-attention uses head dimension $d_{h}=64$

(10) $$A_{h}=\mathrm{softmax}\left(\frac{Q_{h}K_{h}^{⊤}}{\sqrt{d_{h}}}\right)V_{h},\;A=\mathrm{Concat}\left(A_{1},\ldots ,A_{4}\right)W_{O}.$$

Attention dropout is 0.10. The two query outputs are averaged to obtain $\bar{A}\in R^{256}$ and introduced through a second learned gate

(11) $$z=\mathrm{Dropout}[\mathrm{LN}(b+g_{a}\bar{A})],\;g_{a}=\sigma \left(a_{a}\right),$$

where $g_{a}\approx 0.10$ at initialization and the fused-representation dropout rate is 0.25. The resulting $z\in R^{256}$ is provided to both the concept and latent malignancy pathways described in Section 3.3.

#### 3.2.4. Alternative U-Net Morphology Encoder

To assess the influence of the morphology-encoder design, an encoder-only 3D U-Net was evaluated as an alternative to the shared residual 3D CNN. The U-Net comprised five feature levels with 16, 32, 64, 128, and 256 channels. Each level contained two 3×3×3 convolutions followed by batch normalization and ReLU activation, with 2×2×2 max pooling between successive levels. Only the encoder pathway was used; no segmentation decoder was included. Adaptive global-average pooling followed by a linear projection produced the same 256-dimensional feature representation used by the residual-CNN model.

For a controlled comparison, the residual CNN was replaced only by the U-Net encoder. The patient split, local and contextual inputs, context Transformer, gated cross-attention, concept heads, loss functions, augmentation, optimizer, exponential moving average, checkpoint-selection criterion, random seed, and validation-derived threshold rule were kept unchanged.

The comparison used the full BatchNorm U-Net (terminal width 256, learning rate $1\times 10^{-4}$, encoder dropout 0.05, and weight decay $1\times 10^{-3}$) as its reference configuration. A controlled validation-only screen additionally evaluated three compact 192-channel configurations: BatchNorm with learning rate $1\times 10^{-4}$, dropout 0.10, and weight decay $2\times 10^{-3}$; GroupNorm with the same settings; and GroupNorm with learning rate $5\times 10^{-5}$. Screening runs were limited to 12 epochs with an early-stopping patience of four epochs and were ranked using the same gap-penalized validation score as the primary experiment. Only the validation-selected full U-Net was evaluated on the held-out internal test set; losing candidates were not tested.

### 3.3. Hybrid Concept Bottleneck and Loss Function

DS-HCBN uses a hybrid concept bottleneck rather than forcing the complete decision through predefined radiological attributes. Six concepts—subtlety, sphericity, margin, lobulation, spiculation, and texture—are modelled as ordinal 1–5 variables. Internal structure and calcification are categorical with four and six classes. Table 4 summarizes the formulation.

The internal-structure classes correspond to soft tissue, fluid, fat, and air. The six calcification classes are popcorn, laminated, solid, non-central, central, and absent.

#### 3.3.1. Reader-Level Soft Concept Targets

Concept targets were constructed directly from valid individual-reader annotations so that reader disagreement was retained. For an ordinal concept *c*, let $s_{c}^{\left(r\right)}\in \left\{1,\ldots ,5\right\}$ be the rating from reader *r*, with $R_{c}$ valid readers. Each rating is converted to four cumulative indicators,

(12) $$t_{c,k}^{\left(r\right)}=I\left[s_{c}^{\left(r\right)}>k\right],\;k\in \left\{1,2,3,4\right\},$$

and averaged across readers

(13) $$q_{c,k}=\frac{1}{R_{c}}\sum_{r=1}^{R_{c}}t_{c,k}^{\left(r\right)}.$$

The network predicts cumulative probabilities $p_{c,k}=\sigma \left(o_{c,k}\right)$, from which the expected ordinal score is reconstructed as

(14) $${\hat{s}}_{c}=1+\sum_{k=1}^{4}p_{c,k}.$$

For a categorical concept with $K_{c}$ classes, the soft target is the empirical reader distribution,

(15) $$q_{c,j}=\frac{1}{R_{c}}\sum_{r=1}^{R_{c}}I\left[s_{c}^{\left(r\right)}=j\right],\;j=1,\ldots ,K_{c}.$$

The network predicts the corresponding probability vector $\pi_{c}$ with a softmax. Missing or invalid annotations are excluded; when no valid annotation exists for a concept, that concept is masked from the supervision loss rather than imputed.

#### 3.3.2. Hybrid Concept Representation and Malignancy Prediction

The six expected ordinal scores are normalized to [0,1]

(16) $${\tilde{s}}_{c}=\frac{{\hat{s}}_{c}-1}{4},\;c=1,\ldots ,6.$$

Together with the categorical probability vectors for Internal Structure and Calcification, they form the 16-D concept representation

(17) $$c=\left[{\tilde{s}}_{1},\ldots ,{\tilde{s}}_{6};\pi_{\mathrm{IS}};\pi_{\mathrm{Calc}}\right]\in R^{16}.$$

The malignancy head retains two parallel pathways

(18) $$l_{\mathrm{latent}}=f_{\mathrm{latent}}\left(z\right),\;l_{\mathrm{concept}}=f_{\mathrm{concept}}\left(c\right).$$

The final logit is

(19) $$l=l_{\mathrm{latent}}+g_{\mathrm{concept}}l_{\mathrm{concept}},\;g_{\mathrm{concept}}=\sigma \left(a_{\mathrm{concept}}\right),$$

with $g_{\mathrm{concept}}\approx 0.35$ at initialization. The malignancy probability is $P\left(y=1|x_{l},x_{c}\right)=\sigma \left(l\right)$. Because the latent pathway remains available, the concepts are interpreted as radiologically meaningful intermediate representations rather than a complete causal explanation. Their primary supported role is training-time semantic regularization: they supervise representation learning, but neither the architecture nor the intervention results support interpreting the concept pathway as a causal inference mechanism.

#### 3.3.3. Multi-Task Training Objective

Training uses malignancy focal loss, concept supervision, and pairwise ranking

(20) $$L=L_{\mathrm{focal}}+\lambda_{c}L_{\mathrm{concept}}+\lambda_{r}L_{\mathrm{rank}},\;\lambda_{c}=0.35,\;\lambda_{r}=0.10.$$

For label y∈{0,1} and malignancy probability $\hat{y}$, focal loss is

(21) $$L_{\mathrm{focal}}=-\alpha y{\left(1-\hat{y}\right)}^{\gamma }\log\hat{y}-\left(1-\alpha \right)\left(1-y\right){\hat{y}}^{\gamma }\log\left(1-\hat{y}\right),$$

with α=0.60 and γ=1.5. Ordinal concepts use binary cross-entropy over the four cumulative outputs, while categorical concepts use soft-target cross-entropy

(22) $$L_{c}^{\mathrm{ord}}=-\frac{1}{4}\sum_{k=1}^{4}\left[q_{c,k}\log p_{c,k}+\left(1-q_{c,k}\right)\log\left(1-p_{c,k}\right)\right],$$

(23) $$L_{c}^{\mathrm{cat}}=-\sum_{j=1}^{K_{c}}q_{c,j}\log\pi_{c,j}.$$

The concept loss is averaged over valid concept–sample pairs only. A pairwise ranking term encourages malignant samples to receive higher logits than benign samples

(24) $$L_{\mathrm{rank}}=\frac{1}{\left|P\right|}\sum_{(l^{+},l^{-})\in P}\mathrm{softplus}\left[m-\left(l^{+}-l^{-}\right)\right],\;m=0.50.$$

When a mini-batch does not contain both classes, the ranking contribution is set to zero. The independent empirical contribution of these terms is examined in Section 4.5.

### 3.4. Evaluation Protocols for Concept Fidelity and Model Attribution

Concept-based interpretability and image-level attribution were evaluated using the frozen DS-HCBN model. The evaluation comprised three complementary components: fidelity of the predicted radiological concepts, functional influence of the explicit concept pathway on malignancy prediction, and spatial faithfulness of the Grad-CAM attribution maps. No retraining, fine-tuning, checkpoint selection, threshold optimization, or calibration fitting was performed as part of these analyses.

Concept fidelity was evaluated on the held-out LIDC-IDRI test set using metrics suited to the native annotation structure. For the six ordinal concepts, performance was measured using mean absolute error (MAE), quadratic weighted kappa (QWK), Spearman correlation, exact-category agreement, and within-one-category agreement. Internal Structure and Calcification were evaluated using accuracy and, where more than one reference class was represented, balanced accuracy, macro-F1, and Cohen’s kappa. Associations between the ordinal concept scores and malignancy were examined using Spearman correlation and were interpreted descriptively rather than causally.

To determine whether the explicit concept pathway materially influenced malignancy prediction, inference-time concept interventions were performed while keeping the latent representation and all learned model parameters fixed. The predicted 16-dimensional concept vector was replaced by a zero vector, a cohort-mean neutral representation, concept vectors randomly permuted across test cases, or reader-derived concept representations. The permutation intervention was repeated 20 times. The frozen classification threshold of 0.23 was retained throughout, and changes in ROC-AUC, PR-AUC, sensitivity, specificity, F1-score, predicted malignancy probability, and threshold-level decisions were recorded. In addition, aggregate concept error was compared between correctly and incorrectly classified nodules using a one-sided Mann–Whitney test and was related to absolute malignancy-probability error using Spearman correlation.

Spatial attribution was evaluated using three-dimensional Grad-CAM applied to the first, local-input call of the terminal residual encoder block (stage s4 in the implementation). The attribution target was fixed to the original prediction of the frozen model: the malignancy logit was used for cases predicted as malignant and its negative for cases predicted as benign. Grad-CAM maps were upsampled to the local $64^{3}$ input resolution using trilinear interpolation, followed by ReLU and per-case min–max normalization.

Quantitative faithfulness was assessed on a prespecified true-label-stratified subset of 64 nodules from 50 patients, comprising 45 benign and 19 malignant nodules. The subset was selected using random seed 42 before attribution values or perturbation results were examined. Each local $64^{3}$ patch was divided into non-overlapping $8^{3}$ blocks, producing 512 perturbation units that were ranked by their mean Grad-CAM attribution. The $96^{3}$ contextual input remained unchanged throughout the perturbation analysis. Deletion and insertion curves were evaluated over 12 perturbation steps, giving 13 curve points including the unperturbed endpoint. A case-specific smoothed version of the local input, obtained using $17^{3}$ three-dimensional average pooling, served as the perturbation baseline. Each case was also evaluated using 10 matched random block orderings. Lower deletion AUC and higher insertion AUC were prespecified as favorable Grad-CAM behavior.

The beneficial differences were defined as

(25) $$\Delta_{\mathrm{del}}={\mathrm{AUC}}_{\mathrm{random}}-{\mathrm{AUC}}_{\mathrm{GradCAM}},\;\Delta_{\mathrm{ins}}={\mathrm{AUC}}_{\mathrm{GradCAM}}-{\mathrm{AUC}}_{\mathrm{random}}.$$

Positive values therefore favor Grad-CAM for both analyses. Uncertainty was estimated using 10,000 patient-cluster bootstrap replicates. Paired Grad-CAM–random AUC differences were first aggregated at the patient level and then evaluated using one-sided Wilcoxon signed-rank tests in the prespecified favorable direction.

A cascading model-randomization sanity analysis was additionally performed to determine whether the Grad-CAM maps depended on learned model parameters. Starting from the trained model, the prediction heads, fusion and contextual components, encoder stage s4, encoder stage s3, and finally the complete morphology encoder were progressively randomized. The target class remained fixed to the prediction of the trained model. Attribution maps from each randomized model were compared with the corresponding trained-model maps using Spearman correlation on maps downsampled to $16^{3}$ and Jaccard overlap of the top 10% most salient voxels. This analysis was interpreted separately from the deletion/insertion experiment because sensitivity to parameter randomization does not by itself establish perturbation-based spatial faithfulness.

The corresponding concept-fidelity, concept-intervention, Grad-CAM perturbation, and model-randomization results are reported in Section 4.6.

### 3.5. Robustness and Computational Evaluation Protocols

The 1558 nodules with mean malignancy strictly between 2 and 4 were evaluated in an exploratory analysis using the unchanged frozen residual-CNN checkpoint. No retraining, fine-tuning, threshold selection, or relabelling was performed. Because these cases do not have a defensible binary reference label, classification accuracy was not calculated. Instead, model behaviour was summarized using the distribution of malignancy probabilities and Spearman correlation between probability and mean radiologist rating. Confidence intervals were obtained by resampling patients. Results were also stratified by the locked patient split; the uncertain nodules belonging to test-set patients provide the held-out analysis, whereas the full uncertain cohort is descriptive.

Sensitivity to annotation association was evaluated by repeating the constrained complete-linkage grouping at thresholds of 5, 7.5, 10, 12.5, and 15 mm. The 10-mm setting was the reference. For each setting, we recorded eligible nodule and class counts and the proportion of annotation pairs co-clustered at 10 mm that remained co-clustered. This was a data-construction analysis and did not involve model retraining or test-set optimization.

Computational complexity was measured for the frozen residual-CNN DS-HCBN and the supplementary U-Net variant using their trained checkpoints, paired $64^{3}$ and $96^{3}$ inputs, batch size 1, evaluation mode, and automatic mixed precision. After 10 warm-up runs, latency was measured over 50 repetitions on an NVIDIA RTX 3500 Ada Generation Laptop GPU (NVIDIA Corporation, Santa Clara, CA, USA) with PyTorch 2.6.0 and CUDA 12.4. Trainable parameters, peak allocated GPU memory, and PyTorch-profiler FLOPs were recorded. Because not every 3D attention operator exposes FLOP metadata, profiler-counted FLOPs are reported as reproducible lower-bound estimates.

## 4. Experiments and Results

This section evaluates DS-HCBN using the fixed patient-wise protocol described above. The experiments cover internal and external classification performance, calibration and uncertainty, component ablations, and quantitative explainability analyses. The held-out internal test set and LNDb external cohort were evaluated only after the architecture, checkpoint-selection procedure, and primary operating-point rule had been fixed.

### 4.1. Experimental Setup

Experiments used the fixed LIDC-IDRI training, validation, and held-out test partitions reported in Section 3.1. The frozen DS-HCBN architecture contained 3,767,479 trainable parameters. The principal training and evaluation settings are summarized in Table 5.

The checkpoint-selection score was

(26) $$S_{\mathrm{ckpt}}=0.55\;{\mathrm{AUC}}_{\mathrm{val}}+0.45\;{\mathrm{PRAUC}}_{\mathrm{val}}-0.20\;\Delta_{\mathrm{AUC}}^{+}-0.10\;\Delta_{\mathrm{PRAUC}}^{+},$$

where

(27) $$\begin{array}{c} \Delta_{\mathrm{AUC}}^{+}=\max\left({\mathrm{AUC}}_{\mathrm{train}}-{\mathrm{AUC}}_{\mathrm{val}},0\right),\;\Delta_{\mathrm{PRAUC}}^{+}=\max\left({\mathrm{PRAUC}}_{\mathrm{train}}-{\mathrm{PRAUC}}_{\mathrm{val}},0\right). \end{array}$$

**Figure 3:**
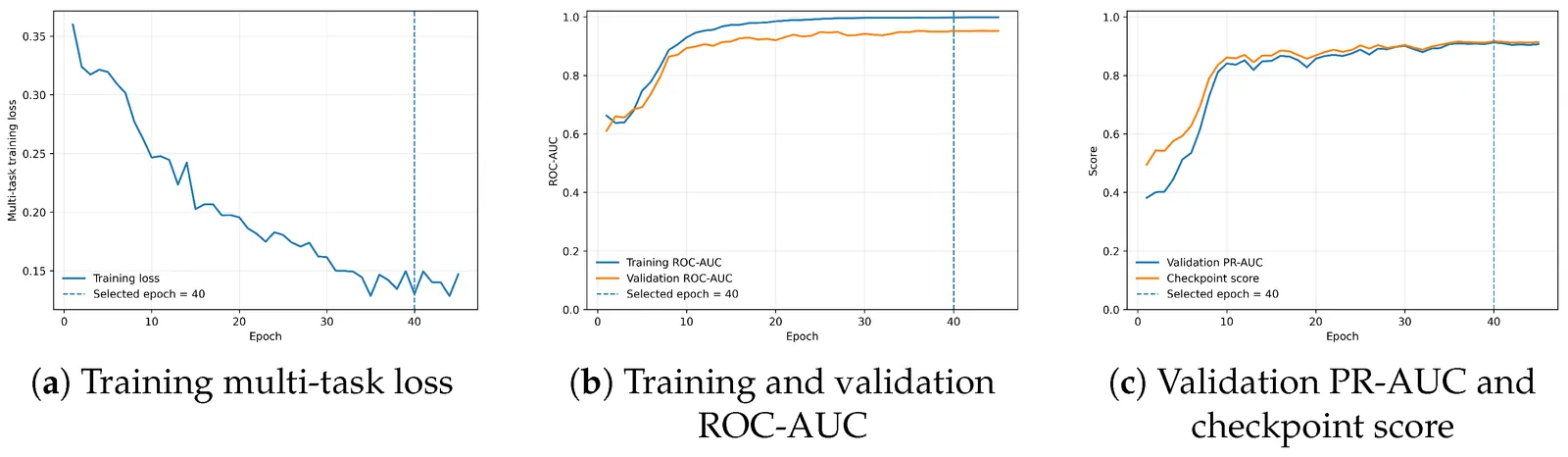
Training and validation behavior of the frozen DS-HCBN model. The dashed vertical marker denotes the selected checkpoint at epoch 40.

The selected checkpoint occurred at epoch 40. The primary operating threshold was then chosen exclusively from validation predictions by maximizing specificity subject to sensitivity ≥0.90. Searching thresholds from 0.01 to 0.99 in increments of 0.005 yielded 0.23. This threshold was frozen before both held-out LIDC-IDRI and LNDb evaluation. No model selection, threshold tuning, calibration fitting, or fine-tuning was performed on either evaluation cohort. The value 0.23 is therefore an experimental operating point rather than a clinically validated decision threshold. The training behavior is shown in Figure 3. These curves are reported for diagnostic transparency and were not used to alter the checkpoint-selection rule.

### 4.2. Evaluation Metrics

Malignant nodules were treated as the positive class. Threshold-dependent performance was measured using accuracy, precision, sensitivity, specificity, F1-score, F2-score, and balanced accuracy. With the usual definitions of TP, TN, FP, and FN, these metrics were calculated as

(28) $$\begin{array}{ccc} \mathrm{Accuracy} & = & \frac{TP+TN}{TP+TN+FP+FN},\\ \mathrm{Precision} & = & \frac{TP}{TP+FP},\\ \mathrm{Sensitivity} & = & \frac{TP}{TP+FN},\\ \mathrm{Specificity} & = & \frac{TN}{TN+FP},\\ F1 & = & \frac{2\;\mathrm{Precision}\;\mathrm{Sensitivity}}{\mathrm{Precision}+\mathrm{Sensitivity}},\\ F2 & = & \frac{5\;\mathrm{Precision}\;\mathrm{Sensitivity}}{4\;\mathrm{Precision}+\mathrm{Sensitivity}},\\ \mathrm{Balanced}\;\mathrm{Accuracy} & = & \frac{\mathrm{Sensitivity}+\mathrm{Specificity}}{2}. \end{array}$$

Threshold-independent discrimination was assessed using ROC-AUC and PR-AUC, with average precision used to summarize precision–recall performance. PR-AUC was included because malignant nodules formed the minority class.

Probability calibration was evaluated using the Brier score,

(29) $$\mathrm{Brier}=\frac{1}{N}\sum_{i=1}^{N}{\left(p_{i}-y_{i}\right)}^{2},$$

where $p_{i}$ is the predicted malignancy probability and $y_{i}\in \left\{0,1\right\}$ is the reference label. Expected calibration error (ECE) was computed from *M* probability bins as

(30) $$\mathrm{ECE}=\sum_{m=1}^{M}\frac{|B_{m}|}{N}\left|{\bar{y}}_{m}-{\bar{p}}_{m}\right|,\;{\bar{p}}_{m}=\frac{1}{|B_{m}|}\sum_{i\in B_{m}}p_{i},\;{\bar{y}}_{m}=\frac{1}{|B_{m}|}\sum_{i\in B_{m}}y_{i}.$$

No post-hoc calibration model was fitted. Sampling uncertainty was estimated using 10,000 cluster-bootstrap replicates. Patients were resampled as clusters for LIDC-IDRI and CT scans as clusters for LNDb, preserving within-subject dependence among nodules. The reported 95% confidence intervals are the 2.5th and 97.5th bootstrap percentiles. Replicates containing only one class were excluded for discrimination measures that require both classes.

### 4.3. Internal and External Validation Results

At the conventional threshold of 0.50, the selected validation checkpoint achieved 87.50% sensitivity and 92.11% specificity. The prespecified high-sensitivity rule yielded the primary threshold of 0.23, increasing validation sensitivity to 91.67% while retaining 89.47% specificity. Table 6 summarizes the two validation operating points.

**Figure 4:**
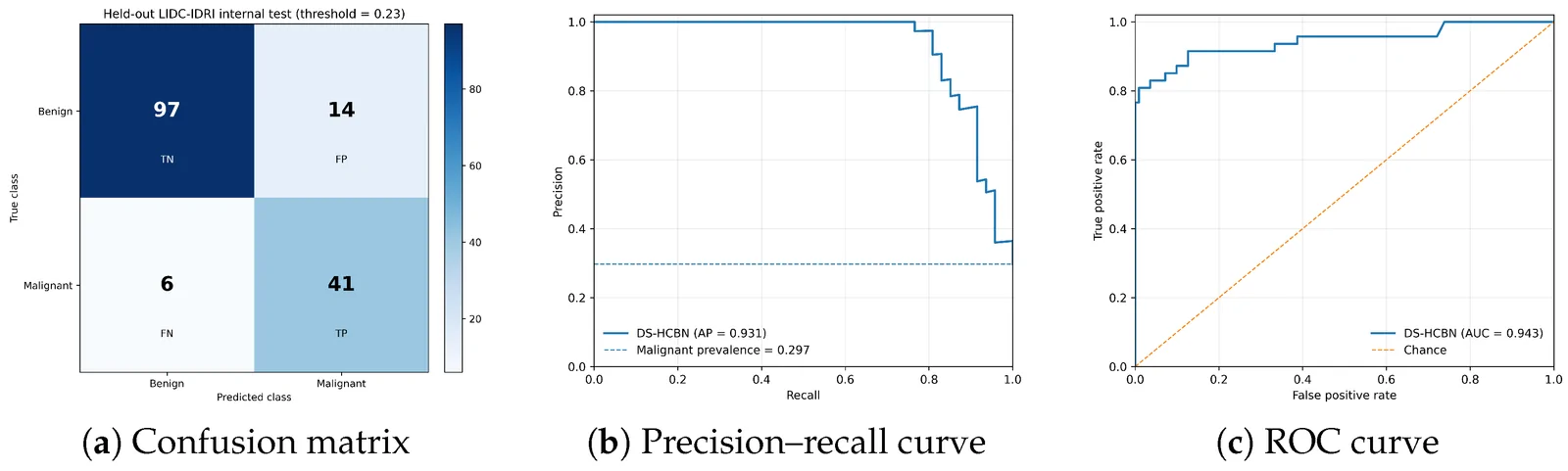
Performance of the frozen DS-HCBN model on the held-out LIDC-IDRI internal test set. The confusion matrix uses the validation-selected threshold of 0.23, whereas the precision–recall and ROC curves summarize threshold-independent discrimination.

Using the unchanged threshold of 0.23, the held-out LIDC-IDRI test set achieved 87.34% accuracy, 87.23% sensitivity, 87.39% specificity, an F1-score of 80.39%, a ROC-AUC of 94.35%, and a PR-AUC of 93.11%. The full set of point estimates is reported in Table 7. Figure 4 shows the internal confusion matrix, precision–recall curve, and ROC curve.

The internal confusion matrix contained 97 true negatives, 14 false positives, 6 false negatives, and 41 true positives. All six false-negative malignant nodules had a mean malignancy score of exactly 4, and four were based on a single malignancy rating. In contrast, all 29 malignant nodules with mean malignancy above 4 were correctly identified. Thus, the remaining false negatives were concentrated among borderline and more weakly supported malignant annotations; this pattern does not imply that the corresponding reference labels were incorrect.

**Figure 5:**
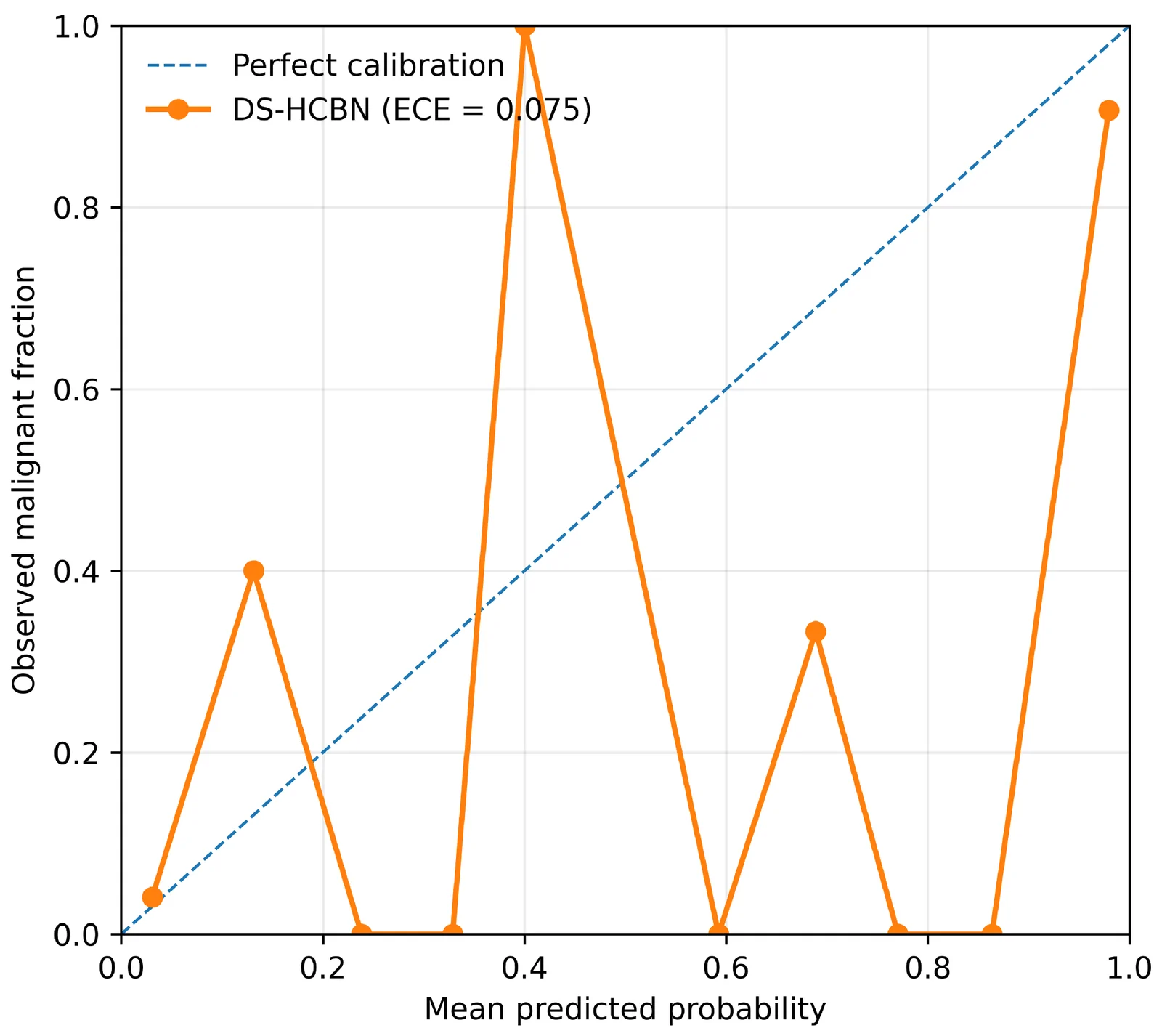
Reliability diagram of the frozen DS-HCBN model on the held-out LIDC-IDRI internal test set. No post-hoc calibration fitting was applied.

Figure 5 complements the Brier and ECE values by showing the relation between predicted malignancy probability and observed malignant frequency.

For external evaluation, the publicly annotated LNDb dataset [36], specifically the partition designated as the training partition in the LNDb release, was used only as an external cohort. The official challenge-test annotations are withheld. LNDb was not used for training, checkpoint selection, threshold optimization, calibration fitting, or fine-tuning. Three entries with malignancy value 0 were treated as invalid and excluded before label harmonization. The same binary rule as LIDC-IDRI was then applied: ratings ≤2 were benign, ratings ≥4 were malignant, and intermediate ratings were excluded as uncertain. The resulting cohort contained 536 binary nodules from 194 CT scans, comprising 462 benign and 74 malignant nodules. At the unchanged threshold of 0.23, external accuracy was 80.41%, sensitivity 63.51%, specificity 83.12%, F1-score 47.24%, ROC-AUC 78.65%, and PR-AUC 54.38%. Table 7 summarizes the complete internal and external point estimates.

Table 8 reports bootstrap uncertainty and calibration. The external reduction is present in ROC-AUC as well as threshold-dependent metrics, so it cannot be explained by the fixed threshold alone. Malignant prevalence was lower in LNDb (13.81%) than in the internal test cohort (29.75%), which contributes to the lower precision and PR-AUC but does not account for the accompanying decline in ROC-AUC.

External false-negative analysis showed a similar pattern to the internal cohort. Of the 27 missed malignant nodules, 24 had a mean malignancy rating of exactly 4 and 20 were supported by a single reader. Together with the reductions in discrimination, sensitivity, and calibration, this finding indicates moderate cross-dataset performance under domain shift rather than established clinical robustness.

### 4.4. Ambiguous-Nodule, Annotation-Threshold, and Complexity Analyses

The frozen primary residual-CNN model was applied to all 1558 uncertain nodules from 686 patients. Predicted malignancy probability was positively associated with the mean radiologist rating (ρ=0.337, patient-bootstrap 95% CI 0.285–0.387; p<0.001). The median probability was 0.065 (IQR 0.027–0.654); 34.0% of cases exceeded the frozen 0.23 operating threshold and 27.6% exceeded 0.50. In the held-out subset comprising 165 uncertain nodules from 64 test-set patients, the association remained positive (ρ=0.272, 95% CI 0.119–0.424; p<0.001). These results indicate that the frozen binary model preserves limited ordinal risk information among ambiguous nodules; they do not establish multiclass accuracy or clinical discrimination among borderline cases. The 10-mm reconstruction exactly reproduced the analysed cohort of 2664 nodules from 875 patients. Within the practically adjacent 7.5–15-mm range, the eligible cohort varied by at most three nodules and co-cluster retention remained at least 99.43%. Among annotation assignments that remained confidently labelled at both settings, no direct benign-to-malignant or malignant-to-benign reversal occurred. The stricter 5-mm threshold produced 25 additional clusters and a larger malignant count, consistent with fragmentation of multi-reader groups; this supports 10 mm as a reasonable central association value while showing that very restrictive grouping is less stable, as shown in Table 9.

The full DS-HCBN contains 3,767,479 trainable parameters. Its profiler-counted complexity, batch-1 inference latency, and peak GPU memory are reported in Table 10. The residual CNN used 20.7% fewer parameters than the U-Net variant, whereas the U-Net had lower measured latency and peak allocated memory on the specified hardware. The similar profiler-counted FLOPs should be interpreted under the stated operator-coverage limitation. Encoder selection was based on the prespecified validation balance and frozen classification results rather than computational speed alone.

### 4.5. Comparative and Ablation Analysis

The proposed model was compared with representative CT-based pulmonary-nodule classification studies. Because the studies differ in cohort composition, label construction, preprocessing, input representation, splitting strategy, and evaluation protocol, the values in Table 11 are contextual rather than a controlled ranking.

#### 4.5.1. Comparison with Published Methods

Several published approaches report higher threshold-dependent accuracy, sensitivity, or specificity under their own protocols. DS-HCBN is therefore not presented as uniformly superior. Its contribution should instead be considered together with the leakage-controlled patient-wise evaluation, radiologist-supervised intermediate concepts, uncertainty and calibration analysis, and external evaluation without adaptation.

#### 4.5.2. Ablation Study

Ablation experiments were conducted using only the fixed LIDC-IDRI training and validation subsets; the held-out internal test set was not used for model-component assessment. Each variant was trained with seed 42 and evaluated using its own validation-selected threshold, obtained by maximizing specificity subject to sensitivity ≥0.90. Table 12 summarizes the results.

The ablations show that local 3D morphology provides the dominant discriminative signal. The local-only model remained close to the full model, whereas removing the Transformer reduced both ROC-AUC and high-sensitivity specificity. The Transformer-only variant performed poorly, indicating that contextual self-attention is most useful as a complementary representation rather than as a stand-alone classifier.

The parameter-matched concatenation baseline contained 3,765,898 parameters, compared with 3,767,479 for the full model, a difference of approximately 0.04%. The full gated cross-attention model achieved higher ROC-AUC, PR-AUC, high-sensitivity specificity, and high-sensitivity F1 than this closely matched baseline. Together with the weaker additive-fusion result, this is consistent with a benefit from gated local-context fusion that is not explained by parameter count alone.

The concept-related ablations show a different pattern. Removing concept supervision reduced ROC-AUC and high-sensitivity F1, suggesting a useful representation-level effect of radiological supervision. However, the latent-only variant performed comparably to the complete model, whereas the concept-only variant remained competitive but weaker on several measures. These results do not show that the concept-derived logit independently improves malignancy classification, which is examined more directly through the intervention analysis in Section 4.6. Removing the pairwise-ranking loss also did not reduce validation performance and is therefore not interpreted as an independently demonstrated source of improvement.

A post-hoc validation-only analysis additionally examined the contextual field of view while keeping the local patch fixed at $64^{3}$. This analysis was performed after the $96^{3}$ design had been fixed and was not used to alter the frozen model, operating threshold, or held-out test results. The comparison is shown in Table 13.

The pre-existing $96^{3}$ context size provided the strongest overall validation balance, with the highest ROC-AUC, high-sensitivity specificity, and high-sensitivity F1, although the $64^{3}$ setting gave a slightly higher PR-AUC. The analysis therefore supports $96^{3}$ as a reasonable context size for the frozen architecture rather than establishing it as universally optimal.

#### 4.5.3. Morphology-Encoder Sensitivity Analysis

The influence of the convolutional morphology encoder was evaluated by replacing the shared residual 3D CNN with an encoder-only 3D U-Net while keeping the remainder of the DS-HCBN architecture and training protocol unchanged. Table 14 presents the validation-only comparison.

The full U-Net achieved a validation ROC-AUC of 94.60% and a PR-AUC of 91.76%, compared with 95.21% and 91.27%, respectively, for the residual-CNN model. Thus, the U-Net produced a slightly higher PR-AUC, whereas the residual CNN produced a slightly higher ROC-AUC. A larger difference was observed at the prespecified high-sensitivity operating point. The residual CNN achieved 89.47% specificity and an F1-score of 84.62%, compared with 71.05% specificity and an F1-score of 70.40% for the full U-Net.

Controlled validation-only hyperparameter screening did not identify a U-Net configuration that improved the overall predefined selection score. Reducing the final U-Net encoder width from 256 to 192 channels lowered the number of trainable parameters from 4.750 million to 3.738 million and improved high-sensitivity specificity to 80.70%. However, its ROC-AUC and PR-AUC decreased to 92.69% and 90.80%, respectively. GroupNorm variants produced lower validation discrimination than the BatchNorm configurations.

The residual CNN was therefore retained as the primary morphology encoder because it provided a more balanced validation operating point and greater parameter efficiency. The U-Net results nevertheless indicate that the choice of morphology encoder can alter the trade-off between malignant-nodule sensitivity and false-positive classification.

Table 15 presents the secondary internal-test comparison. On the held-out internal test cohort, the U-Net achieved higher threshold-independent discrimination, increasing ROC-AUC from 94.35% to 97.90% and PR-AUC from 93.11% to 96.31%. It also increased sensitivity from 87.23% to 97.87%, reducing the number of false-negative malignant nodules from six to one. However, the number of false positives increased from 14 to 26, resulting in lower specificity, precision, accuracy, and F1.

These findings demonstrate a clinically relevant trade-off. The U-Net favored malignant-nodule sensitivity and threshold-independent discrimination, whereas the residual CNN provided a more balanced operating point. Because the model-selection criterion was defined on the validation cohort and emphasized both discrimination and generalization, the residual CNN remained the primary DS-HCBN encoder. Because this comparison was performed after the primary residual-CNN architecture and evaluation protocol had been fixed, it is treated as a secondary exploratory analysis. The internal-test findings were not used to retune either encoder or to redefine the primary architecture.

### 4.6. Concept Fidelity, Intervention, and Attribution Results

This section reports the concept-fidelity, concept-intervention, and spatial- attribution results obtained using the prespecified evaluation protocols described in Section 3.4.

Table 16 summarizes the prediction performance of the six ordinal radiological concepts. Concept fidelity varied across attributes. Subtlety showed the strongest rank agreement with the reader-derived targets, followed by Spiculation and Lobulation, whereas Sphericity and Texture showed weaker agreement.

For the categorical concepts, only one Internal Structure reference class was represented in the internal test cohort. The resulting 100% accuracy therefore reflects performance only for the observed class and does not establish four-class discrimination. For Calcification, four of the six possible classes were represented; accuracy was 89.87%, balanced accuracy 44.93%, macro-F1 29.26%, and Cohen’s κ=0.713. The difference between overall and class-balanced performance reflects the strong class imbalance among the observed calcification categories.

The reader-derived ordinal concepts were also associated with malignancy. The strongest positive correlations were observed for Lobulation (ρ=0.601), Spiculation (ρ=0.594), and Subtlety (ρ=0.567), whereas Sphericity, Margin, and Texture showed negative associations; all correlations had p≤0.012. These associations are descriptive and do not imply that individual concepts have an independent causal effect on the malignancy prediction.

Because concept accuracy alone does not show whether the classifier actually uses the explicit concept pathway, inference-time interventions were performed while keeping the latent representation and model parameters unchanged. Table 17 summarizes the results.

The interventions produced only very small changes in malignancy probability and caused no prediction flips at the frozen threshold. This indicates that the explicit concept-to-logit pathway has limited direct influence on the final frozen prediction, which is dominated by the latent imaging representation. However, removing concept supervision during training reduced validation performance in Table 12, suggesting that the concepts still provide useful representation-level supervision.

Concept quality was also related to classification difficulty. Mean aggregate concept error was 0.182 for incorrectly classified nodules compared with 0.130 for correctly classified nodules (Mann–Whitney U=1904, p=0.0031), and aggregate concept error correlated with absolute malignancy-probability error (ρ=0.317, p<0.001). These findings indicate an association between poorer concept prediction and more difficult malignancy cases, without implying a causal relationship.

**Figure 6:**
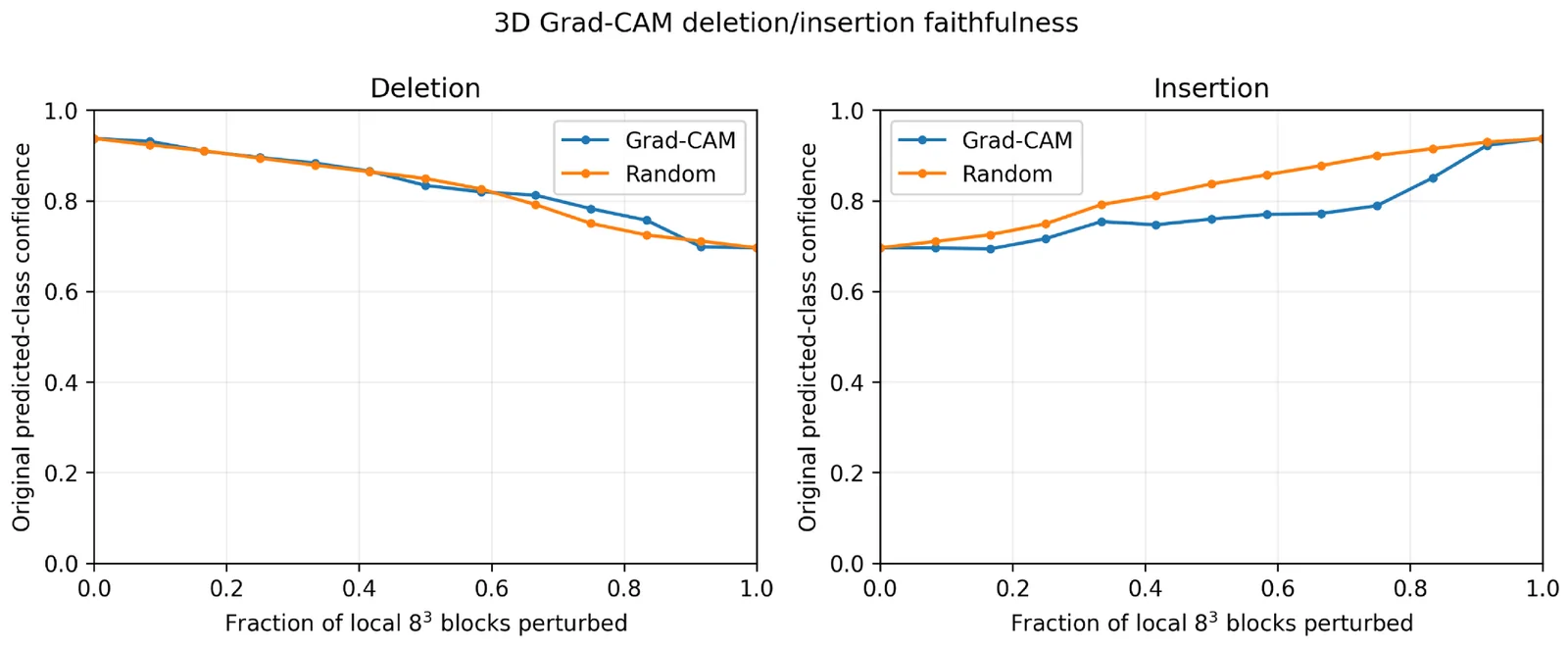
Deletion and insertion faithfulness analysis for 3D Grad-CAM on the prespecified held-out LIDC-IDRI subset. Grad-CAM-ranked perturbation ordering is compared with matched random block ordering.

Under the prespecified deletion/insertion protocol described in Section 3.4, Grad-CAM-ranked perturbations were compared with matched random block orderings. Figure 6 shows the resulting curves, and Table 18 reports the corresponding quantitative comparisons.

Grad-CAM did not outperform matched random ordering for either deletion or insertion. In particular, insertion performance was less favorable than the random baseline. Thus, the prespecified perturbation analysis did not establish spatial faithfulness for the Grad-CAM attribution maps.

**Figure 7:**
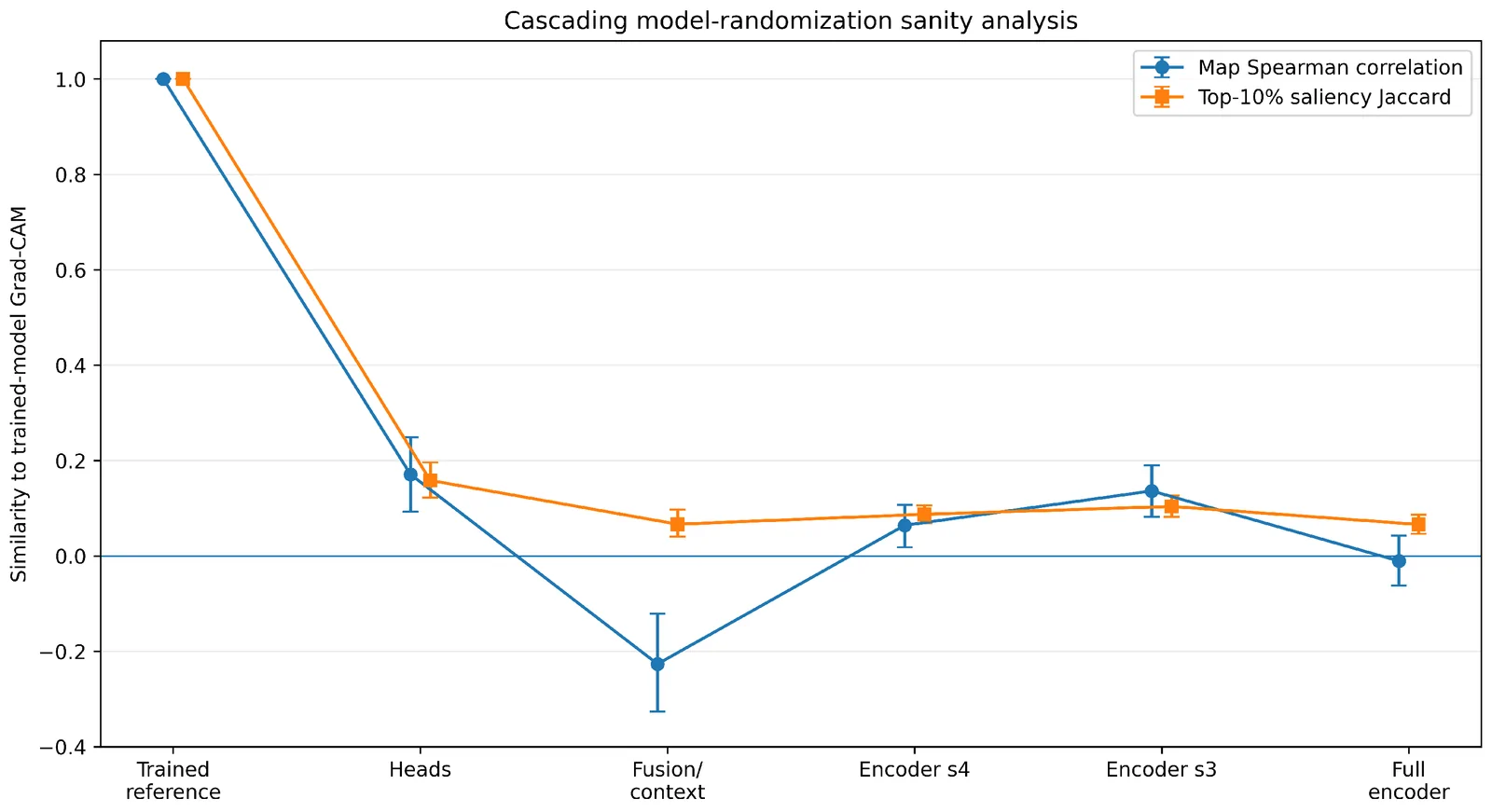
Cascading parameter-randomization sanity analysis for 3D Grad-CAM. Spearman correlation and top-region Jaccard overlap compare attribution maps from the trained model with maps obtained after progressive randomization of learned parameters.

A complementary parameter-randomization analysis was used to determine whether the attribution maps depended on learned model parameters. Figure 7 summarizes this analysis.

Attribution similarity became low after randomization of substantial portions of the network. Following full encoder randomization, the mean Spearman correlation with the trained-model maps was −0.011 and the mean top-region Jaccard overlap was 0.066. The changes across intermediate randomization stages were not monotonic. The results therefore indicate that the attribution maps depend on learned model parameters, but parameter sensitivity alone does not establish spatial faithfulness.

Taken together, the explainability analyses support a limited and quantitative interpretation of the model. DS-HCBN predicts radiological intermediate concepts with measurable but heterogeneous fidelity, while the explicit concept pathway has only a small direct effect on frozen malignancy predictions. Grad-CAM is sensitive to learned parameters but did not demonstrate perturbation-based faithfulness relative to matched random ordering. DS-HCBN is therefore best interpreted as a hybrid concept-guided classifier with evaluated intermediate representations rather than as a strict concept-mediated or fully faithful explanatory model.

## 5. Discussion

The leakage-controlled evaluation shows strong internal discrimination but also clarifies where the model remains uncertain. On the held-out LIDC-IDRI test set, DS-HCBN achieved a ROC-AUC of 94.35% and a PR-AUC of 93.11%, with 87.23% sensitivity and 87.39% specificity at the validation-selected threshold of 0.23. The six false-negative malignant nodules all had a mean malignancy score of 4, and four had only a single malignancy rating. The errors therefore concentrated near the binary decision boundary rather than among the more clearly malignant nodules.

The ablation results indicate that local 3D morphology supplies most of the discriminative signal, while contextual modelling is complementary. The local-only model remained close to the full model, whereas the Transformer-only variant was weak. The parameter-matched concatenation baseline was also weaker than gated cross-attention despite an almost identical parameter count. These results are consistent with a modest benefit from content-dependent local-context interaction rather than from additional capacity alone. The post-hoc context-size analysis similarly supports $96^{3}$ context as a reasonable overall validation setting, although it was not optimal for every metric. The concept analyses require a cautious interpretation. Several radiological concepts were predicted with moderate agreement to reader annotations, and larger concept error was associated with larger malignancy-prediction error. However, latent-only classification was comparable to the complete model, and inference-time concept interventions produced only minimal probability changes and no prediction flips. Concept supervision therefore appears to help primarily at the representation level, while the explicit concept residual has little direct effect on the frozen decision. DS-HCBN is consequently better described as a hybrid concept-guided model than as a strict concept bottleneck.

The spatial attribution results support the same bounded view. Grad-CAM maps changed after parameter randomization, showing dependence on learned model parameters, but they did not outperform matched random ordering in the deletion/insertion experiment. Insertion performance was worse than the random baseline. The maps are therefore interpreted strictly as post-hoc sensitivity visualizations, not as causal spatial explanations of individual predictions.

External evaluation on LNDb revealed a clear domain-shift limitation of the current model. Using the unchanged checkpoint and threshold, ROC-AUC fell to 78.65% and sensitivity to 63.51%, with poorer calibration. Lower malignant prevalence in LNDb contributes to the decline in precision and PR-AUC, but it does not explain the reduction in ROC-AUC. Differences in acquisition and reconstruction, nodule characteristics, annotation practice, and reader support are plausible contributors. Internally, most external false negatives were malignancy-4 and single-reader cases. These findings do not establish robustness across institutions or acquisition domains.

The morphology-encoder sensitivity analysis showed that the residual CNN and encoder-only U-Net produced different performance profiles. The U-Net achieved higher internal-test ROC-AUC, PR-AUC, and sensitivity and reduced false-negative malignant nodules from six to one. However, it generated 12 additional false positives and produced lower specificity, precision, accuracy, and F1 at its validation-selected threshold.

The residual CNN also required fewer trainable parameters, containing 3.767 million parameters compared with 4.750 million for the full U-Net. In addition, the U-Net selected an early checkpoint and showed declining validation performance during subsequent training, suggesting greater susceptibility to overfitting in the present dataset. Controlled normalization, channel-width, learning-rate, and regularization screening did not identify a U-Net configuration that surpassed the residual CNN’s overall validation balance.

These results do not indicate that one encoder is universally superior. The U-Net may be preferable in applications that assign a particularly high cost to false-negative malignant nodules. In contrast, the residual CNN provides a more balanced and parameter-efficient solution when both sensitivity and specificity are considered. On this basis, the residual CNN was retained as the primary morphology encoder.

Some limitations remain. The model is strictly a classifier of pre-localized nodules: it requires an externally supplied centroid and is not an end-to-end nodule detector or localizer. Intermediate malignancy ratings remain outside the primary binary endpoint. Although the exploratory frozen-model analysis demonstrated modest ordinal risk association in ambiguous nodules, it does not establish multiclass performance or clinical utility for borderline cases. Concept labels are reader-derived and subject to inter-observer variability, and neither the concept nor Grad-CAM explanations have been independently assessed by radiologists. The encoder comparison used a single training seed, and the U-Net internal-test analysis was secondary to the prespecified evaluation of the primary residual-CNN model. External evaluation was limited to one public cohort and showed substantial domain shift. The annotation-threshold and computational analyses reduce two methodological uncertainties, but broader threshold validation, hardware-independent profiling, multiple training seeds, and additional untouched external cohorts remain necessary.

## 6. Conclusions and Future Work

This study presented DS-HCBN, a dual-scale hybrid concept-guided network for malignancy classification of pre-localized pulmonary nodules in 3D CT. The framework combines local and contextual volumetric information through a shared residual 3D CNN, a context-only Transformer, gated cross-attention, and supervision from eight radiological concepts. Under the leakage-controlled patient-wise protocol, DS-HCBN achieved 87.34% accuracy, 87.23% sensitivity, 87.39% specificity, an F1-score of 80.39%, a ROC-AUC of 94.35%, and a PR-AUC of 93.11% on the held-out LIDC-IDRI test set. Ablation analysis showed that local morphology provides the dominant signal, while contextual modelling and gated cross-attention contribute complementary information.

The interpretability analyses support a limited rather than absolute claim. Radiological concepts were predicted with measurable but heterogeneous fidelity, but interventions on the concept representation had little direct effect on frozen malignancy predictions. Concept supervision is therefore supported primarily as training-time semantic regularization rather than a causal inference mechanism. Grad-CAM was sensitive to learned parameters but did not demonstrate perturbation-based spatial faithfulness relative to matched random ordering and is interpreted strictly as a post-hoc sensitivity visualization. The model is therefore best viewed as a hybrid concept-guided classifier rather than a fully concept-mediated or causally explainable system.

The morphology-encoder analysis showed that an encoder-only 3D U-Net increased internal-test discrimination and sensitivity but reduced specificity and overall operating-point balance. Validation-only hyperparameter screening did not identify a U-Net configuration that provided a stronger overall validation profile. The shared residual 3D CNN was therefore retained as the primary encoder because it provided a more balanced and parameter-efficient solution.

External evaluation on LNDb yielded 80.41% accuracy and a ROC-AUC of 78.65%, with reduced sensitivity and calibration relative to LIDC-IDRI. Frozen-model analysis of ambiguous nodules showed a modest positive association between predicted risk and reader rating, while annotation grouping was stable across 7.5–15 mm. These additions do not broaden the primary binary endpoint. Future work should prioritize multi-centre external validation, improved domain generalization and acquisition harmonization, explicit modelling of borderline and uncertain malignancy labels, more strongly intervenable concept pathways, independent radiologist assessment of concept and spatial explanations, and integration with automatic nodule detection and localization.

## Figures and Tables

**Table 1:** Comparison of representative methods most closely related to the proposed DS-HCBN. Reported results are reproduced from the corresponding studies and are not directly comparable because datasets and evaluation protocols differ.

Study	Representation/Dataset	Reported Performance	Main Distinction from DS-HCBN
Huang et al. [21]	3D self-supervised learning with domain adaptation; LIDC-IDRI	Acc. 91.07%; AUC 95.84%	Focuses on representation transfer; no explicit radiological concept pathway or local-context fusion.
Yang et al. [22]	Multi-view 3D CNN with squeeze-and-excitation; LIDC-IDRI	Acc. 96.04%; Sens. 98.59%	Uses complementary 3D views, whereas DS-HCBN explicitly separates local morphology and larger contextual volumes.
Yu et al. [23]	3D Faster R-CNN + 3D BCDU-Net; LIDC-IDRI/LUNA16	Acc. 91.50%; F1 90.63%	Addresses a broader 3D detection/segmentation-oriented pipeline; DS-HCBN assumes pre-localized nodules and focuses on malignancy classification.
Sun et al. [25]	CT-image and radiological-attribute representation with Transformer; LIDC-IDRI	Acc. 90.60%; Rec. 92.81%	Uses attributes in multimodal contrastive learning; DS-HCBN predicts concepts as intermediate outputs within the malignancy pathway.
Liu et al. [24]	Handcrafted morphology/texture + CNN features; LIDC-IDRI	Acc. 91.21%; F1 91.04%	Combines engineered and learned features but lacks dual-scale contextual modelling and an explicit concept bottleneck.
Wang et al. [33]	Multi-task malignancy and manifestation prediction with anatomical attention; LIDC-IDRI + in-house data	AUC 0.992/0.923	Provides semantic auxiliary predictions, but not the same hybrid concept-bottleneck and gated local-context fusion formulation.
Rafferty et al. [34]	Expert-guided concept model; chest radiography	Multiple metrics reported	Closely related conceptually, but differs in imaging modality and does not address 3D CT nodule-level dual-scale modelling.

**Table 2:** Malignancy label assignment based on the mean radiologist malignancy score.

Mean Malignancy Score	Final Label
≤2	Benign (0)
>2 and <4	Uncertain; excluded from binary classification
≥4	Malignant (1)

**Table 3:** Final patient-wise stratified-random split of the binary LIDC-IDRI cohort. Counts refer to original nodules; no augmented samples are included.

Subset	Patients	Benign	Malignant	Total Nodules
Training	412	568	218	786
Validation	88	114	48	162
Internal test	89	111	47	158
Total	589	793	313	1106

**Table 4:** Radiological concept formulation used in DS-HCBN.

Radiological Concept	Annotation Type	Prediction Formulation
Subtlety	Ordinal, 1–5	4 cumulative logits
Sphericity	Ordinal, 1–5	4 cumulative logits
Margin	Ordinal, 1–5	4 cumulative logits
Lobulation	Ordinal, 1–5	4 cumulative logits
Spiculation	Ordinal, 1–5	4 cumulative logits
Texture	Ordinal, 1–5	4 cumulative logits
Internal structure	Categorical, 4 classes	4-class softmax
Calcification	Categorical, 6 classes	6-class softmax

**Table 5:** Experimental setup and principal hyperparameters of the frozen DS-HCBN model.

Component	Setting
**Development dataset**	LIDC-IDRI
**External evaluation**	Publicly annotated LNDb partition used only as an external evaluation cohort
**Local/context input**	$64^{3}$/$96^{3}$ voxels at 1-mm isotropic spacing
**Morphology encoder**	Shared residual 3D CNN with GroupNorm and SiLU
**Latent representation**	256 dimensions
**Context Transformer**	Context only; $8^{3}$ patch embedding; dimension 128; depth 2; 4 heads; MLP ratio 2
**Fusion**	Local-anchored gated cross-attention with 2 local query tokens
**Concept formulation**	6 ordinal concepts + internal structure (4-class) + calcification (6-class)
**Classifier**	Hybrid latent logit + learned gated concept logit
**Trainable parameters**	3,767,479
**Optimizer**	AdamW, $\beta_{1}=0.9$, $\beta_{2}=0.999$
**Initial learning rate**	$1\times 10^{-4}$
**Weight decay**	$1\times 10^{-3}$
**Batch size**	2
**Maximum epochs**	45
**Warm-up/scheduler**	3 epochs/cosine decay
**Early-stopping patience**	8 epochs
**EMA decay**	0.995
**Gradient clipping**	1.0
**Dropout**	Classifier 0.25; encoder 0.05; Transformer 0.15
**Class balancing**	Weighted sampling on the training subset only
**Train-only augmentation**	Paired local/context flips and $90^{\circ }$ rotations; intensity shift ±0.015; Gaussian noise σ≤0.010
**Malignancy loss**	Focal loss: positive-class α=0.60, γ=1.5
**Concept-loss weight**	$\lambda_{c}=0.35$
**Pairwise-ranking weight/margin**	$\lambda_{r}=0.10$; margin =0.50
**Random seed**	42
**Checkpoint selection**	Validation ROC-AUC/PR-AUC score with positive train–validation-gap penalties
**Primary threshold rule**	Highest validation specificity subject to sensitivity ≥0.90; search grid 0.01–0.99 in steps of 0.005
**Frozen primary threshold**	0.23
**Calibration fitting**	None
**Software/hardware**	PyTorch 2.7.1+cu118; NVIDIA RTX 3500 Ada Generation Laptop GPU (12 GB; NVIDIA Corporation, Santa Clara, CA, USA); CUDA automatic mixed precision (AMP)

**Table 6:** Validation-set operating points used to document threshold selection for the frozen DS-HCBN model. All values except the threshold are percentages. Boldface identifies the validation-selected primary operating point determined using the prespecified high-sensitivity threshold-selection rule.

Threshold	Acc.	Prec.	Sens.	Spec.	F1
0.50	90.74	82.35	87.50	92.11	84.85
**0.23**	**90.12**	**78.57**	**91.67**	**89.47**	**84.62**

**Table 7:** Primary internal and external performance of the frozen DS-HCBN model at the unchanged threshold of 0.23 (%).

Cohort	N	Acc.	Prec.	Sens.	Spec.	F1	F2	AUC	PR-AUC
LIDC-IDRI internal test	158	87.34	74.55	87.23	87.39	80.39	84.36	94.35	93.11
LNDb external cohort	536	80.41	37.60	63.51	83.12	47.24	55.82	78.65	54.38

**Table 8:** Bootstrap uncertainty and calibration of the frozen DS-HCBN model.

Cohort	AUC 95% CI	PR-AUC 95% CI	Sens. 95% CI	Spec. 95% CI	Brier	ECE
LIDC-IDRI internal test	88.60–98.54	86.23–97.69	76.32–95.92	80.39–93.46	0.0866	0.0751
LNDb external cohort	72.00–85.38	42.63–66.92	52.17–75.00	78.92–87.10	0.1298	0.1096

**Table 9:** Sensitivity of LIDC-IDRI annotation association to the maximum centroid-separation threshold. Co-cluster retention is relative to annotation pairs grouped together at the 10-mm reference.

Threshold (mm)	Eligible	Benign	Uncertain	Malignant	Co-Cluster Retention (%)
5.0	2689	794	1558	337	99.08
7.5	2663	791	1556	316	99.61
10.0	2664	793	1558	313	100.00
12.5	2665	795	1560	310	99.69
15.0	2667	797	1558	312	99.43

**Table 10:** Computational characteristics of the primary residual-CNN DS-HCBN and supplementary U-Net encoder variant. Latency is median (IQR) over 50 batch-1 mixed-precision repetitions.

Encoder	Parameters	Profiler GFLOPs	Latency (ms)	Peak GPU Memory (MB)
Residual CNN	3,767,479	1.595	44.16 (42.24–46.14)	168.84
U-Net encoder	4,750,231	1.589	36.06 (34.24–38.38)	147.03

**Table 11:** Contextual comparison with representative published lung-nodule classification studies. Values are reported as percentages under the evaluation protocols of the respective studies. Differences in cohort construction, label aggregation, data partitioning, and threshold selection preclude direct statistical ranking across methods; “–” indicates that the corresponding metric was not reported for the comparable setting.

Study	Method	Acc.	Sens.	Spec.	F1	AUC
Yang et al. [22]	3D-MVSECNN	96.04	98.59	90.00	–	94.30
Sun et al. [25]	Nodule-CLIP	90.60	92.81	92.60	–	–
Yu et al. [23]	3D-NoduleNet	91.50	92.50	90.79	90.63	–
Liu et al. [24]	Mixed-feature model	91.21	90.27	91.98	91.04	–
Moreno et al. [3]	3D multi-attention (binary; LIDC-IDRI)	–	91.60	–	–	93.95
**DS-HCBN**	**Dual-scale hybrid concept model**	87.34	87.23	87.39	80.39	94.35

**Table 12:** Validation-only component ablations at seed 42. HS Spec. and HS F1 denote specificity and F1-score at each variant’s highest-specificity validation threshold satisfying sensitivity ≥90%. All variants reached 91.67% sensitivity at the reported high-sensitivity operating point.

Variant	Params. (M)	AUC	PR-AUC	HS Spec.	HS F1
Full model reference	3.767	95.21	91.27	89.47	84.62
Local only	2.845	94.54	90.78	86.84	82.24
No Transformer	2.845	93.33	90.26	82.46	78.57
Transformer only	0.591	61.46	44.59	21.93	48.62
Parameter-matched concatenation	3.766	94.39	87.80	84.21	80.00
Additive fusion	3.209	92.79	87.95	80.70	77.19
Latent only	3.767	95.29	91.17	91.23	86.27
Concept only	3.767	94.37	88.83	80.70	77.19
No concept supervision	3.767	90.63	84.07	72.81	71.54
No pairwise ranking loss	3.767	95.30	91.45	89.47	84.62

**Table 13:** Post-hoc validation-only context-size sensitivity analysis at seed 42, with the local patch fixed at $64^{3}$.

Context Size	AUC	PR-AUC	HS Spec.	HS F1
$64^{3}$	93.19	91.62	75.44	73.33
$80^{3}$	93.12	88.05	71.05	70.40
$96^{3}$	95.21	91.27	89.47	84.62
$112^{3}$	93.27	89.83	69.30	69.29

**Table 14:** Validation-only morphology-encoder sensitivity analysis at seed 42. HS Spec. and HS F1 denote specificity and F1-score at the highest-specificity validation threshold satisfying sensitivity ≥90%. Boldface indicates the highest value in each performance-metric column.

Encoder Configuration	Params. (M)	AUC	PR-AUC	HS Spec.	HS F1
Shared residual CNN	3.767	**95.21**	91.27	**89.47**	**84.62**
Full U-Net, BatchNorm	4.750	94.60	**91.76**	71.05	70.40
Compact U-Net, BatchNorm	3.738	92.69	90.80	80.70	77.19
Compact U-Net, GroupNorm	3.738	91.43	85.35	77.19	74.58
Compact U-Net, GroupNorm, LR $5\times 10^{-5}$	3.738	88.54	83.08	50.88	59.46

**Table 15:** Secondary internal-test comparison of morphology encoders. Each encoder was evaluated using its independently frozen validation-selected threshold. All values except the threshold are percentages. Boldface indicates the highest value in each performance-metric column.

Encoder	Thr.	Acc.	Prec.	Sens.	Spec.	F1	F2	AUC	PR-AUC
Residual CNN	0.23	**87.34**	**74.55**	87.23	**87.39**	**80.39**	84.36	94.35	93.11
Encoder-only U-Net	0.22	82.91	63.89	**97.87**	76.58	77.31	**88.46**	**97.90**	**96.31**

**Table 16:** Prediction performance for the six ordinal radiological concepts on the held-out LIDC-IDRI internal test set. QWK denotes quadratic weighted kappa and ρ denotes Spearman rank correlation.

Concept	MAE	QWK	ρ	Exact (%)	Within 1 (%)
Subtlety	0.658	0.573	0.698	48.73	87.97
Sphericity	0.661	0.191	0.278	46.84	93.67
Margin	0.775	0.377	0.491	45.57	84.18
Lobulation	0.635	0.416	0.511	62.03	91.14
Spiculation	0.574	0.516	0.549	64.56	91.14
Texture	0.663	0.231	0.349	61.39	84.18

**Table 17:** Inference-time concept interventions on the held-out LIDC-IDRI internal test set. The classification threshold remained fixed at 0.23. |Δp| denotes the mean absolute change in predicted malignancy probability relative to normal inference.

Condition	AUC	PR-AUC	Sens.	Spec.	F1	|Δp|
Normal inference	94.35	93.11	87.23	87.39	80.39	0.00000
Zero concepts	94.36	93.17	87.23	87.39	80.39	0.00247
Mean-neutral concepts	94.36	93.17	87.23	87.39	80.39	0.00022
Permuted concepts	94.34	93.14	87.23	87.39	80.39	0.00032
Reader-derived concepts	94.36	93.17	87.23	87.39	80.39	0.00031

**Table 18:** Quantitative Grad-CAM deletion/insertion faithfulness results. For deletion, a positive beneficial difference corresponds to lower Grad-CAM AUC than random ordering; for insertion, it corresponds to higher Grad-CAM AUC than random ordering. Confidence intervals were obtained using patient-cluster bootstrap resampling.

Analysis	Grad-CAM AUC	Random AUC	Beneficial Difference (95% CI)	One-Sided *p*
Deletion	0.834	0.828	−0.0055 [−0.0332,0.0206]	0.638
Insertion	0.774	0.827	−0.0528 [−0.0871,−0.0224]	0.940

## Data Availability

The datasets used in this study are publicly available. The LIDC-IDRI dataset can be obtained from The Cancer Imaging Archive (TCIA) [35], and the LNDb dataset can be obtained from its official public repository [36]. No new clinical dataset was created in this study. The implementation supporting this study will be publicly available in the DS-HCBN GitHub repository at https://github.com/absargana/DS-HCBN (accessed on 13 September 2026).
